# Notch signaling regulates myogenic regenerative capacity of murine and human mesoangioblasts

**DOI:** 10.1038/cddis.2014.401

**Published:** 2014-10-09

**Authors:** M Quattrocelli, D Costamagna, G Giacomazzi, J Camps, M Sampaolesi

**Affiliations:** 1 Translational Cardiomyology Lab, Stem Cell Institute Leuven, Department of Development and Regeneration, KU Leuven, Belgium; 2 Division of Human Anatomy, University of Pavia, Pavia, Italy

## Abstract

Somatic stem cells hold attractive potential for the treatment of muscular dystrophies (MDs). Mesoangioblasts (MABs) constitute a myogenic subset of muscle pericytes and have been shown to efficiently regenerate dystrophic muscles in mice and dogs. In addition, HLA-matched MABs are currently being tested in a phase 1 clinical study on Duchenne MD patients (EudraCT #2011-000176-33). Many reports indicate that the Notch pathway regulates muscle regeneration and satellite cell commitment. However, little is known about Notch-mediated effects on other resident myogenic cells. To possibly potentiate MAB-driven regeneration *in vivo*, we asked whether Notch signaling played a pivotal role in regulating MAB myogenic capacity. Through different approaches of loss- and gain-of-function in murine and human MABs, we determined that the interplay between Delta-like ligand 1 (Dll1)-activated Notch1 and Mef2C supports MAB commitment *in vitro* and ameliorates engraftment and functional outcome after intra-arterial delivery in dystrophic mice. Furthermore, using a transgenic mouse model of conditional *Dll1* deletion, we demonstrated that Dll1 ablation, either on the injected cells, or on the receiving muscle fibers, impairs MAB regenerative potential. Our data corroborate the perspective of advanced combinations of cell therapy and signaling tuning to enhance therapeutic efficaciousness of somatic stem cells.

Notch signaling consists of a conserved pathway, triggered by physical interaction between one ligand and one receptor, both transmembrane proteins exposed by contacting cells.^1^ Notch signaling has been involved in different stages of muscle formation^2^ and regeneration.^3,4^ The canonical signaling encompasses five ligands (Dll1/3/4, Jagged1/2) and four receptors (Notch1–4); however, the axis Dll1-Notch1 appears consistently involved during myogenic fate specification, for example, neural crest-driven somite maturation.^5^ Moreover, murine embryos expressing a hypomorphic allele of the Notch ligand *Dll1* displayed marked impairment of skeletal muscle formation.^6^ Interestingly, the Notch pathway may exert different effects according to the cell context. Culture on DLL1-coated plastic improved *ex vivo* proliferation and *in vivo* engraftment of canine satellite cells.^7^ Expression of the active *Notch1* intracellular domain (*NICD*) robustly committed murine and rat mesenchymal stem cells toward the myogenic fate both *in vitro* and *in vivo*.^8^ However, Notch-mediated effects on the regenerative potential of non-satellite resident myogenic cells are still unknown.

Mesoangioblasts (MABs) are non-satellite resident myogenic stem cells, able to circulate and regenerate dystrophic skeletal muscles.^9,10^ HLA-matched MABs are currently under phase 1 clinical study on Duchenne muscular dystrophy patients (EudraCT #2011-000176-33). In this view, understanding the cell-specific effects and mechanisms of myogenic cues will help improving clinical translation of MAB-based therapies *in vivo*. Recently, it has been shown that Notch synergizes with Pdgf-bb to convert fetal myoblasts into myogenic pericytes.^11^ However, knowledge about Notch-triggered effects on the regenerative potency of somatic MABs is still scant, particularly in the contexts of cell–cell (*in vitro*) and fiber–cell (*in vivo*) contact.

Therefore, we asked whether the Dll1-Notch1 axis regulates the myogenic potential of murine and human MABs and how to tune this pathway to ameliorate *in vivo* MAB-driven regeneration.

## Results

### Dll1-Notch1 regulate *in vitro* differentiation of murine and human MABs

**Figure 1:**
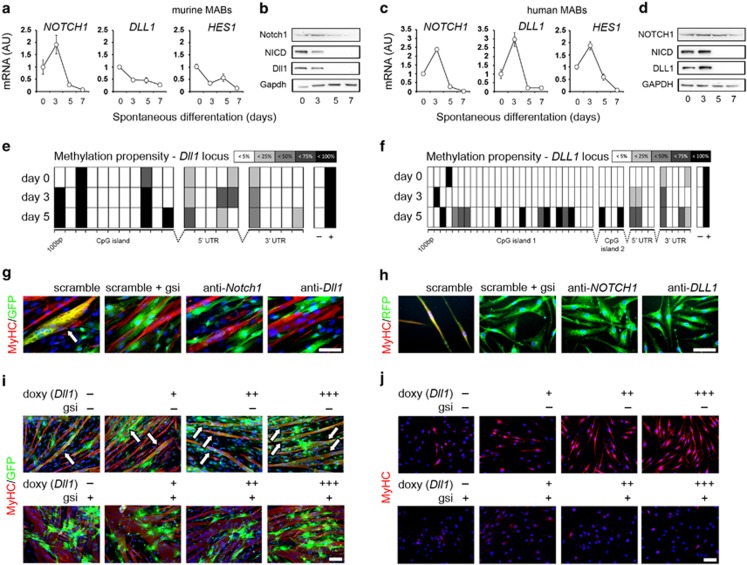
The axis Dll1-Notch1 regulates the *in vitro* myogenic potential of murine and human MABs. (**a–d**) Expression levels of *Notch1/NOTCH1, Dll1/DLL1* and *Hes1/HES1* (Notch1 activation reporter), and WB analysis of Notch1/NOTCH1, its activated form (Notch1/NICD) and Dll1/DLL1 in murine and human MABs over time during spontaneous differentiation *in vitro*. Expression levels are reported in arbitrary units (AU) as fold change *versus* day 0. (**e, f**) Summary array of methylation propensity along upstream CpG islands (one in the murine locus, two in the human), 5′-UTR and 3′-UTR of *Dll1/DLL1* genomic loci in murine and human MABs at day 0, day 3 and day 5 of spontaneous differentiation, as assayed by qPCR-based test on genomic fragments enriched in highly methylated DNA. −, unmethylated control, APC promoter; +, methylated control and propensity reference, NBR2 promoter. (**g**) Immunofluorescence staining on co-cultures of C2C12 myoblasts with murine MABs transduced with lentiviral vectors carrying a GFP tracer and scramble, or anti-*Notch1*, or anti-*Dll1* interfering shRNAs. White arrows indicate chimeric GFP^+^/MyHC^+^ myotubes and MAB myogenic differentiation. Scramble and scramble +*γ-*secretase inhibitor (gsi) conditions represent the controls of unperturbed and chemically inhibited signaling, respectively. (**h**) Immunofluorescence staining on differentiated human MABs after transduction with lentiviral vectors carrying a RFP tracer and scramble, or anti-*NOTCH1*, or anti-*DLL1* interfering shRNAs. Presence of MyHC^+^ mono/bi-nucleated myocytes indicate MAB myogenic differentiation. (**i, j**) Immunofluorescence staining to evaluate the dose-dependent effects of transient *Dll1/DLL1* overexpression on the *in vitro* myogenic differentiation of murine and human MABs, both transduced with lentiviral vectors carrying conditional expression of the ligand under doxycycline (doxy) control; −, 0 *μ*g/ml, basal control; +, 0.1 *μ*g/ml; ++, 1 *μ*g/ml; +++, 10 *μ*g/ml. To confirm Notch signaling involvement in the observed effect, gsi-supplemented control conditions are also shown. To assess the myogenic contribution of murine MABs in co-culture with C2C12, GFP^+^ murine MABs have been used in this experiment. Data in charts are depicted as mean±standard deviation of ≥3 independent experiments. Scale bars indicate 100 *μ*m

For this study, we used murine^12^ and human MABs,^13^ both isolated from somatic muscles as alkaline phosphatase^+^ (AP^+^) cells and processed as previously reported^14^ (Supplementary Figure 1). Consistently, with antecedent reports, AP^+^ murine MABs do not spontaneously undergo myogenic differentiation *in vitro*, unlike human AP^+^ MABs^9,10^ (Supplementary Figure 2). We analyzed the pattern of *Notch1*, *Dll1* and *Hes1* expression and of pathway activation during spontaneous *in vitro* differentiation. Murine MABs showed slight upregulation of *Notch1* at day 3, but progressive downregulation of *Dll1 and Hes1* over time, matching Dll1 and NICD (activated receptor) protein levels (Figures 1a and b; Supplementary Figure 3a). Conversely, human MABs showed early upregulation of *NOTCH1, DLL1* and *HES1*, paralleled by increasing protein levels of DLL1 and NICD at day 3, followed by rapid decrease (Figures 1c and d; Supplementary Figure 3a). To investigate the role of epigenetics in the species-specific pattern of *Dll1* expression, we monitored the DNA methylation propensity along the regulatory regions of the genomic *Dll1/DLL1* locus. Interestingly, *Dll1* regulatory regions appeared more rapidly methylated in murine than in human MABs (Figures 1e and f), being the overall methylation propensity along the murine sequences significantly higher at day 0 and 3 than in the human *DLL1* locus (Supplementary Figure 3b). Thus, during spontaneous differentiation *in vitro*, murine MABs do not spontaneously mature into myocytes and exhibit rapid locus methylation and decreased expression of *Dll1* at early stage, correlating with decreased Notch1 activation. Conversely, human MABs mature into myocytes *in vitro* and show sustained *Dll1* expression and Notch1 activation at early differentiation step.

We then investigated the effects of loss and gain of Notch signaling on the *in vitro* myogenic capacity of MABs. This was assayed in murine MABs by co-culture/fusion with C2C12 myoblasts, whereas human MABs were assayed in spontaneous differentiation toward myocytes. Specific knockdown of *Notch1* and *Dll1* (Supplementary Figure 4a), as well as treatment with *γ*-secretase inhibitor (gsi), resulted in decreased NICD levels (Supplementary Figure 4b), and in impairment of murine MAB fusion with myoblast-derived myotubes, as compared with scramble control (Figure 1g). Analogously, gsi and *NOTCH1/DLL1* knockdown led to impaired maturation of human MABs into MyHC^+^ myocytes (Figure 1h). We also analyzed the involvement of other receptors and ligands, for example, Notch2 receptor and Jag1 ligand, in our *in vitro* system. Knockdown of *Notch2/NOTCH2* or *Jag1/JAG1* did not significantly alter MAB myogenic differentiation, as compared with scramble control (Supplementary Figure 5), supporting the hypothesis that the Dll1-Notch1 axis is pivotal in our experimental system. We next used a lentiviral system of doxycycline-triggered *Dll1/DLL1* overexpression to evaluate the effects of gain-of-function in the early differentiation step. After application of doxycycline till day 3 of differentiation, *Dll1/DLL1* overexpression dose-dependently increased *in vitro* myogenic maturation of both murine and human MABs (Figures 1i and j). This effect was significantly decreased in the presence of gsi (Supplementary Figure 6), thus confirming the direct involvement of Dll1-triggered Notch signaling in the early differentiation step of murine and human MABs.

**Figure 2:**
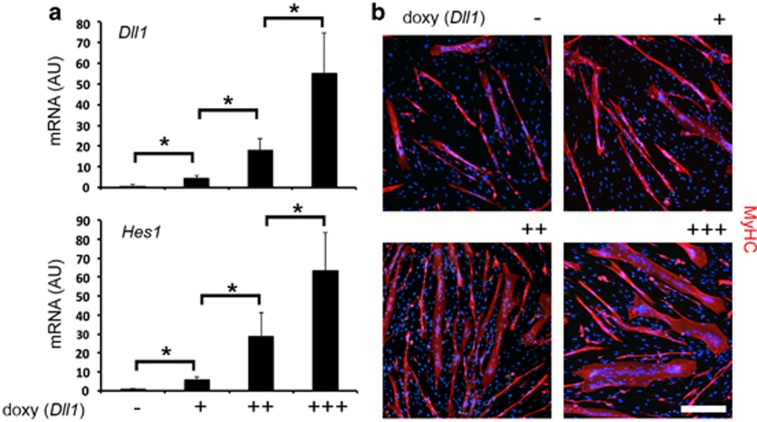
*Dll1* dose-dependently enhances *in vitro* myogenic ability of *Sgcb-null* cardiac MABs. (**a**) According to increasing concentrations of doxycycline, expression levels of *Dll1* and *Hes1* are significantly upregulated at 72 h post transduction (*P<0.05; *n*=6). Expression levels are reported in arbitrary units (AU) as fold change *versus* transduced MABs in absence of doxycycline. Data in charts are depicted as mean±standard deviation. (**b**) Immunofluorescence staining at day 7 of differentiation shows enhanced *in vitro* myogenic ability, following increasing concentrations of doxycycline, applied till day 3. Scale bars indicate 100 *μ*m

As additional proof of principle, we sought to apply our dose-dependent system of transient Dll1 overexpression to a MAB model of robust myogenic differentiation. To this purpose, we used murine *Sgcb-null* MABs, previously reported as aberrantly and spontaneously myogenic.^15,16^ Transient overexpression of *Dll1* dose-dependently correlated with increased *Hes1* expression at day 3 (Figure 2a) and enhanced myotube formation at day 7 (Figure 2b). Thus, Dll1-Notch1 signaling during the early differentiation/commitment step sustains MAB myogenic differentiation at a later stage.

**Figure 3:**
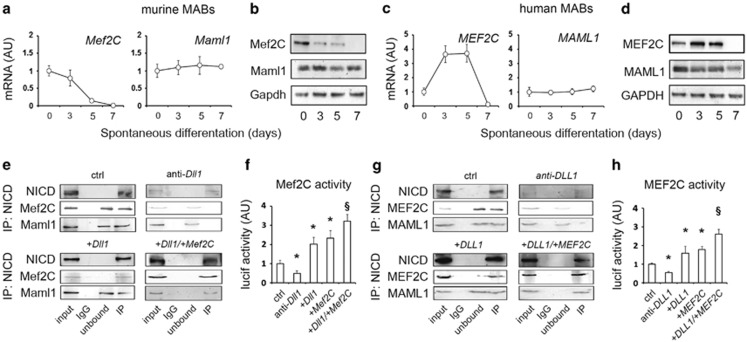
Dll1-triggered Notch signaling in MABs interplays with Maml1 and Mef2C and results in increased Mef2C activity. (**a–d**) Expression and protein levels of *Mef2C/MEF2C* and *Maml1/MAML1* in murine and human MABs over time during spontaneous differentiation *in vitro*. Expression levels are reported in arbitrary units (AU) as fold change *versus* day 0. (**e**) Results of NICD-based immunoprecipitation (IP-NICD) at day 3 of differentiation of murine MABs in control conditions (ctrl), and in conditions of *Dll1* knock-down (anti-Dll1), *Dll1* overexpression (+*Dll1*), and combined overexpression of *Dll1* and *Mef2C (+Dll1/+Mef2C*). Input, protein extract; IgG, mouse IgG-based IP, negative control; unbound, unbound fraction after IP. (**f**) Luciferase activity assayed at day 5 of differentiation after transfection with a plasmid carrying firefly luciferase expression under the control of a Mef2C-responsive element into murine MABs after *Dll1* silencing (*anti-Dll1*), *Dll1* overexpression *(+Dll1*), *Mef2C* overexpression *(+Mef2C*), and combined overexpression of *Dll1/DLL1* and *Mef2C/MEF2C (+Dll1/+Mef2C*). *P<0.05 *versus* ctrl; ^§^*P*<0.05 *versus +Dll1/+DLL1* and *+Mef2C/MEF2C* (*n*=4). Depicted in (**g, h**) are the analogous results obtained with human MABs. Luciferase activity levels are reported in AU as fold change of renilla-normalized values *versus* ctrl conditions. Control cells were transduced with scramble and overexpression vectors and kept doxycycline-free. Data in charts are depicted as mean±standard deviation of ≥3 independent experiments. Scale bars indicate 100 *μ*m

We asked whether *Mef2C* and *Maml1*, reported interactors of NICD in other myogenic cell models,^17,18^ were involved in *Dll1*-dependent effects on MAB differentiation. Murine MABs showed progressive decline of gene and protein levels of *Mef2C* over time, whereas *MEF2C* was upregulated at days 3–5 in human MABs. Moreover, the levels of *Maml1/MAML1* appeared stable over time in both murine and human MABs (Figures 3a and d and Supplementary Figure 7a). Notably, at day 3 of spontaneous differentiation, Mef2C and Maml1 are only partially associated to NICD and *Dll1* knockdown resulted in major dissociation from the residual NICD levels. Conversely, *Dll1* overexpression led to complete Mef2C recruitment, whereas only after combined overexpression of *Dll1* and *Mef2C* did Maml1 appear completely associated to the NICD complex (Figure 3e). Because Maml1 was reported as activator of Mef2C upon dissociation from NICD,^17^ we quantified the effects of Maml1 recruitment on Mef2C activity in our cell system at day 5 of differentiation, when NICD levels are depleted in normal conditions. Using a *Mef2C*-responsive luciferase system, Mef2C transcriptional activity in murine MABs was decreased after *Dll1* knockdown and significantly increased after combined overexpression of *Dll1* and *Mef2C* (Figure 3f). We obtained analogous data also from human MABs (Figure 3g and h). Thus, the Dll1-Notch1 axis appears as a major regulator of the *in vitro* myogenic ability of both murine and human MABs, and Dll1-triggered signaling cooperates with Mef2C and Maml1 in enhancing Mef2C activity at later stages.

### Priming murine and human MABs for enhanced *in vivo* regeneration

**Figure 4:**
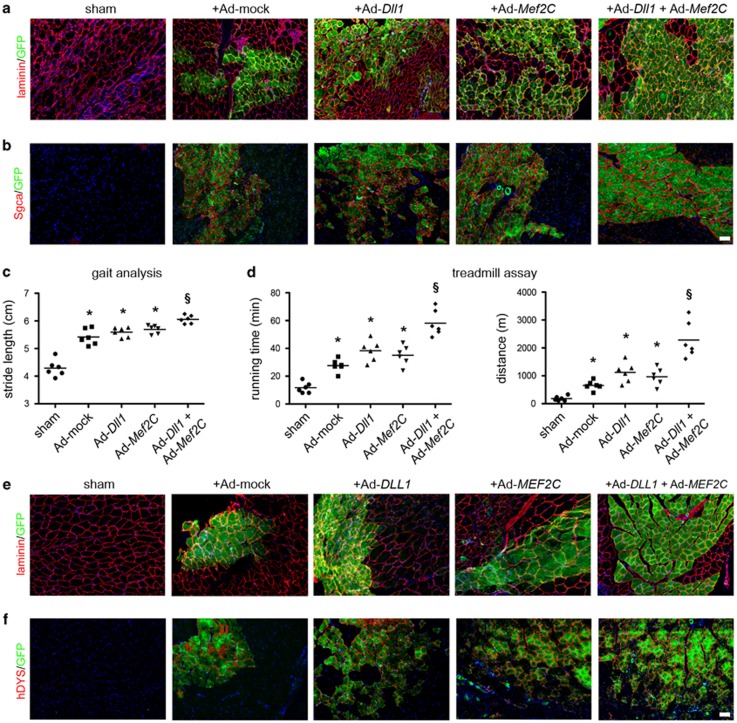
Combined priming with *Dll1/DLL1* and *Mef2C/MEF2C* enhances the *in vivo* regenerative capacity of both murine and human MABs. (**a, b**) Immunofluorescence staining on *gastrocnemius* muscle slides of *Sgca-null* mice at 4 weeks after bilateral femoral artery injection of adenoviral-primed GFP^+^ murine MABs. Laminin/GFP staining (**a**) has been used to evaluate engraftment and Sgca/GFP staining (**b**) for regeneration capacity. (**c, d**) Functional assessment of the adenoviral-based priming on the murine MAB-driven muscle regeneration of dystrophic muscles at 4 weeks post injection. The gait analysis (**c**) has been performed on the stride length of mice spontaneously walking along a 1 m path. The treadmill assay (**d**) has been performed on mice running on a 10°-uphill oriented treadmill belt with 1 m/min^2^ acceleration on 10 m/min starting speed. Data points depict the value of each assayed mouse, bars indicate the average values; *P<0.05 *versus* sham; ^§^*P*<0.05 *versus* Ad*-Dll1* and *versus* Ad-*Mef2C* (*n*=6 mice/group). (**e, f**) Immunofluorescence staining on *gastrocnemius* muscle slides of *Rag2-null/γc-null* mice at 4 weeks after bilateral femoral artery injection of adenoviral-primed GFP^+^ human MABs. Laminin/GFP staining (**e**) has been used to evaluate engraftment and hDYSTROPHIN (hDYS)/GFP staining (**f**) for regeneration capacity. Sham, vehicle-injected control mice; +Ad-mock, MABs primed with Ad-LacZ, adenoviral-treated control cells with unperturbed Notch signaling. Scale bars indicate 100 *μ*m

Subsequently, we sought to determine whether tuning the signaling without genomic integrations could ameliorate the *in vivo* regenerative potential of murine and human MABs. We used an adenoviral-based, non-integrative system of temporary overexpression of *Dll1* or *Mef2C* (Ad-*Dll1* and Ad-*Mef2C*, respectively, Supplementary Figures 7b and c), priming the cells *in vitro* prior to bilateral intra-arterial injection into dystrophic mice. After injection into *Sarcoglycanα(Sgca)-null* mice, combined priming of green fluorescent protein (GFP)^+^ murine MABs with Ad-*Dll1* and Ad-*Mef2C* resulted in enhanced levels of engraftment into GFP^+^ chimeric fibers and restoration of Sgca, as shown by immunostaining, western blotting (WB) and quantitative polymerase chain reaction (qPCR) at 4 weeks (Figures 4a and b and Supplementary Figure 8) and 8 weeks post injection (data not shown). Accordingly, fibrotic scars appeared also reduced in treated mice (Supplementary Figure 9a). We then assessed the functional relevance of histological and molecular data by gait analysis and treadmill assay. In both tests, combined priming of injected MABs significantly ameliorated the functional outcome, both at 4 (Figures 4c and d) and 8 weeks (Supplementary Figure 9b) post injection. Moreover, combined priming did not increase off-target engraftment, which appeared poor in all conditions (Supplementary Figure 10 and data not shown). Comparably, we tested the adenoviral priming system on human MABs and assessed their *in vivo* myogenic potential by bilateral intra-arterial delivery in acutely damaged muscles of *Rag2-null/γc-null* immunodeficient mice.^19^ Similarly to murine MABs, combined priming of human GFP^+^ MABs with Ad-*DLL1* and Ad-*MEF2C* resulted in higher levels of engraftment (Figure 4e) and detection of human-specific DYSTROPHIN and sarcomeric ACTININ*α* (Figure 4f and Supplementary Figure 11). Hence, non-integrative priming with *Dll1* and *Mef2C* enhances myogenic capacity and regenerative potential of both murine and human MABs *in vivo*.

### Analysis of Dll1 requirement using transgenic MABs and muscles

**Figure 5:**
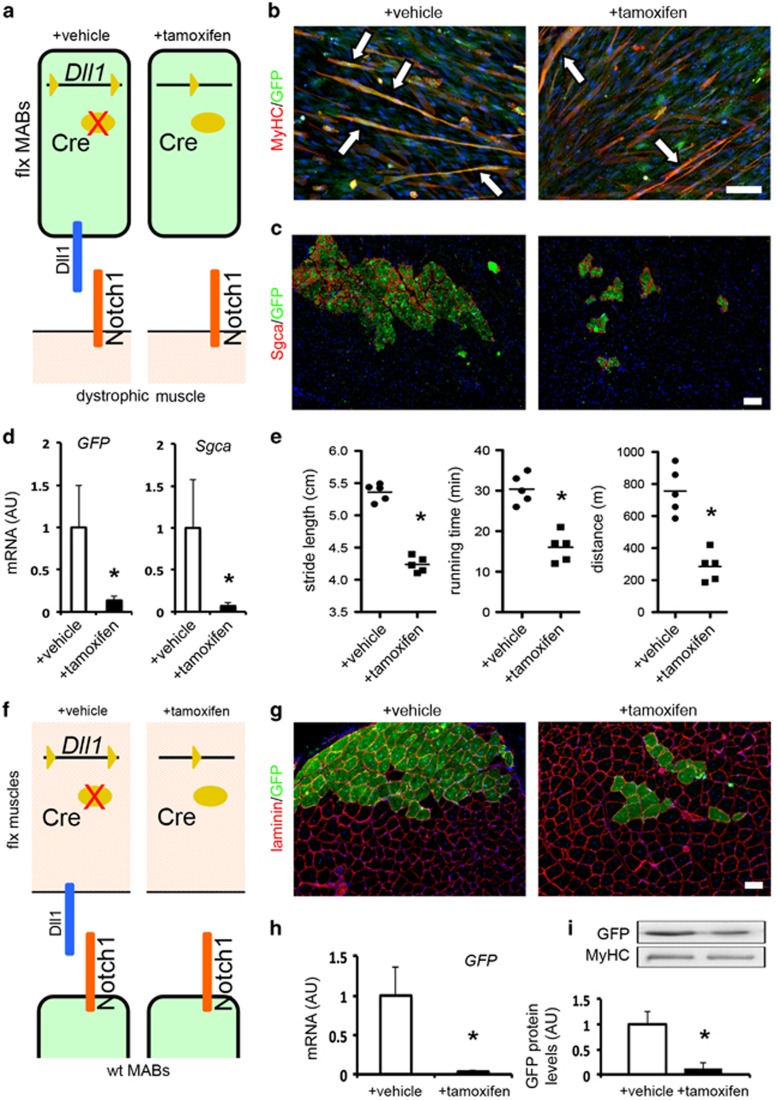
Analysis of Dll1 requirement for MAB engraftment/regenerative capacity using *Dll1*^*flx/flx*^*;Rosa26::iCreERT2*^*+/−*^ (flx) transgenic MABs and muscles. (**a**) Representative scheme of the experiment assessing tamoxifen-driven *Dll1* knockout in the homing flx MABs, using the vehicle-treated cells as control. (**b**) Immunofluorescence staining on co-cultures of C2C12 myoblasts with GFP^+^ flx MABs after *Dll1* knockout. White arrows indicate chimeric MyHC^+^ myotubes and MAB myogenic differentiation. (**c**) Immunofluorescence staining on *gastrocnemius* muscle slides to evaluate the regenerative capacity of *Dll1*-knockout GFP^+^ flx MABs at 8 weeks after intra-arterial injection into *Sgca-null* mice. (**d**) qPCR-based quantification of engraftment (*GFP*) and regeneration (*Sgca*) into the *tibialis anterior* muscles at 8 weeks post injection; *P<0.05 *versus* vehicle-treated flx MABs (*n*=5 mice/group). (**e**) Functional assessment of conditional *Dll1* knockout on MAB-driven regeneration of dystrophic muscles at 8 weeks after bilateral injection, by means of gait analysis (stride length) and treadmill assay (running time, distance). Data points depict the value of each assayed mouse, bars indicate the average values; *P<0.05 *versus* vehicle-treated flx MABs (*n*=5 mice/group). (**f**) Representative scheme of the experiment assessing tamoxifen-driven Dll1 depletion in the flx muscles, using the vehicle-treated mice as control. (**g**) Immunofluorescence staining on *gastrocnemius* muscle slides to evaluate the engraftment ability of homing GFP^+^ MABs into *Dll1*-knockout muscles at 4 weeks after intra-arterial injection. (**h**, **i**) Expression and protein level analyses of GFP to quantify reduced homing ability of MABs into *tibialis anterior* fibers after conditional *Dll1* knockout at 4 weeks post injection; *P>0.05 (*n*=3 mice/group). Expression and protein levels in (**d**, **h**, **i**) are reported in arbitrary units (AU) as fold change *versus* vehicle-treated mice. Scale bars indicate 100 *μ*m

To further examine the directionality of the ligand presentation at the interface between the homing cell and the host fiber, we examined MABs and muscle fibers of *Dll1*^*flx/flx*^*;Rosa26::iCreERT2*^*+/−*^ (flx) transgenic mice, allowing conditional tamoxifen-driven *Dll1* knockout (Supplementary Figures 12a and c). To examine the requirement of Dll1 presentation by the homing cell, we isolated and characterized flx MABs (Supplementary Figures 12d and e) from post-natal muscles of the transgenic mice, as previously described.^14^ We then tested their myogenic potential *in vitro* by fusion with C2C12-derived myotubes, and *in vivo* by intra-arterial delivery into *Sgca-null* mice (Figure 5a). After tamoxifen-driven *Dll1* knockout, flx GFP^+^ MABs showed significant impairment in the contribution to GFP^+^ myotubes *in vitro* (Figure 5b) and to Sgca^+^/GFP^+^ fibers *in vivo* (Figures 5c and d and Supplementary Figures 13a and b), as compared with vehicle-treated cells. Interestingly, *Dll1* knockout in homing flx MABs resulted also in significant reduction of the functional outcome at 4 and 8 weeks post injection, as assayed by gait analysis and treadmill test (Figure 5e and Supplementary Figure 13c). To validate our transgenic model, we also assayed flx satellite cells, which, as expected,^20^ showed reduced levels of Pax7 and increased spontaneous differentiation after tamoxifen addition (Supplementary Figures 14a and b). Subsequently, we asked whether Dll1 presentation by the host fiber is also required. We therefore tested the engraftment levels of wild-type GFP^+^ MABs after intra-arterial delivery into *Dll1*-knockout adult flx muscles (Figure 5f). In the absence of acute damage, tamoxifen-treated mice did not present significant alterations or lesions in skeletal muscles, as compared with vehicle-treated controls (Supplementary Figure 14c). In *Dll1*-knockout muscles, the rate of MAB engraftment was significantly reduced as compared with vehicle-treated controls, as shown by immunostaining, qPCR and WB (Figures 5g and i). Thus, Dll1 presentation by both the homing cell and the host fiber appears a positive regulator of MAB commitment and engraftment.

## Discussion

**Figure 6:**
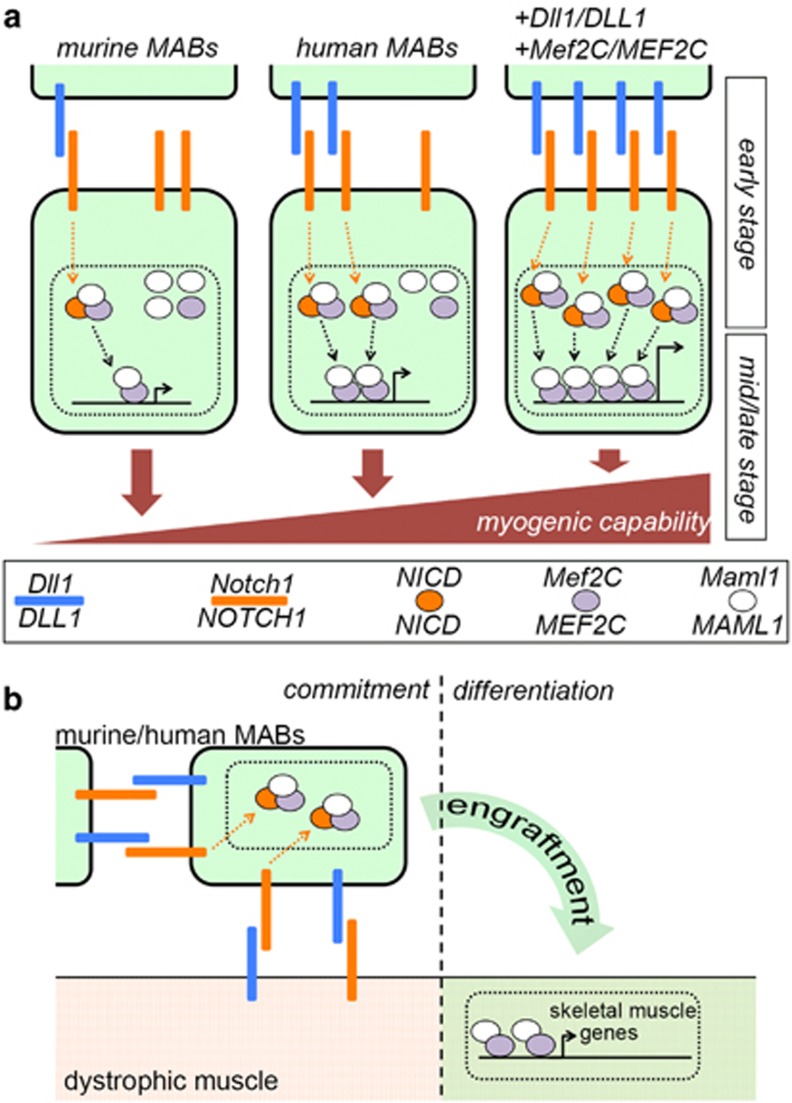
Hypothetical models of Notch-based tuning of MAB myogenic differentiation. (**a**) The intrinsic difference in the levels of Dll1-triggered Notch1 activation and of Mef2C could explain, at least partially, the difference in spontaneous myogenic capability between murine and human MABs in our experimental system. According to this hypothesis, murine MABs present low levels of Dll1-triggered signaling and of Mef2C during the commitment stage. At a later step of differentiation, once NICD is removed from the complex, Mef2C activity levels are therefore minimal. Human MABs present intrinsically higher levels of *DLL1* and *MEF2C in vitro*. As a consequence, murine MABs fail to spontaneously produce mature myocytes *in vitro*, whereas human MABs differentiate, although with low efficiency, spontaneously. Transient overexpression of *Dll1/DLL1* and *Mef2C/MEF2C* during the commitment stage induces complete recruitment of Mef2C/MEF2C and Maml1/MAML1 by NICD, thereby reinforcing Mef2C activity and myogenic capability of both murine and human MABs, as observed *in vivo*. (**b**) Representative scheme of the hypothetical model regarding Dll1 involvement during the commitment-differentiation transition of MABs *in vivo*, according to our experimental setup with transgenic MABs and muscles. Dll1-Notch1 interactions between the dystrophic fiber and the homing MABs trigger their commitment and engraftment, plausibly through Maml1/Mef2C interplay. After engraftment, the signaling is discontinued, NICD removed and the engrafted MABs activate the myogenic program, likely through sustained levels of Mef2C activity

Taken altogether, our data suggest that Dll1-triggered Notch1 activation plays a pivotal role in the myogenic commitment of post-natal murine and human MABs. The difference in *Dll1* epigenetic/transcriptional regulation and in Notch1 activation during the early *in vitro* differentiation step could account, at least partially, for the intrinsic difference in spontaneous *in vitro* myogenic capacity between murine and human MABs. Moreover, Dll1-triggered positive influence on MAB differentiation seems confirmed in the proof of principle of *Dll1* overexpression with the aberrantly myogenic *Sgcb-null* MABs. The aforementioned observations, in combination with the IP and luciferase analyses at days 3 and 5 of differentiation, suggest a time-dependent threshold effect of Dll1-activated NICD interplay with Mef2C and Maml1 (Figure 6a). According to this hypothetical model, in the absence of Dll1-presenting myoblasts, murine MABs exhibit low levels of Dll1 and Mef2C at early differentiation stage and, therefore, Mef2C activity at later stages is likely insufficient to corroborate further progression. Differently, human MABs present sufficient levels of DLL1 and MEF2C to trigger MEF2C activity and myocyte maturation at later stage. Furthermore, in both murine and human MABs, transient overexpression of *Dll1/DLL1* and *Mef2C/MEF2C* during the early stage supposedly saturates complex formation and correlates with enhanced myogenic capacity.

This *in vitro* model is reinforced by our adenoviral-based system of MAB priming prior to injection. Adenoviruses do not integrate in the genome^21^ of the engrafting MABs, still promoting the overexpression pulse of the transgenes and enhancing the commitment. Once transduced cells are engrafted, the vector is likely diluted in the fibers, discontinuing the signaling and ameliorating the regeneration outcome of MABs in targeted muscles.

The directionality of Dll1 requirement for MAB homing and differentiation *in vivo* constitutes an interesting question, considering that Notch signals have been directly linked to the niche engraftment by satellite cells.^22^ We addressed this point by means of a transgenic system of conditional *Dll1* deletion. Together with the data previously discussed, the results obtained from flx MABs and flx muscles suggest a potential, yet incomplete, model of the basal role of Notch on MAB engraftment and differentiation *in vivo* (Figure 6b). According to this model, Notch1 activation in the homing MABs by Dll1-exposing cells or fibers may result in NICD-mediated recruitment of Mef2C and Maml1. Once engrafted in the fiber, potentially also through Dll1-mediated interactions, NICD levels would decrease in the absence of receptor stimulation and Mef2C would then exert its transcriptional activity, promoting the expression of mid-late myogenic factors. Noteworthy, this model might also point to Notch-based recruitment of MABs by Dll1-exposing activated satellite cells^23^ in the context of muscle injury.

Our study cannot exclude the contribution of other ligands or receptors of the Notch family, probably accounting for the remaining engraftment of *Dll1*-knockout MABs or in Dll1-depleted muscles. However, other interactions appeared less significant than the Dll1-Notch1 axis in our experimental setup, considering that knockdown of *Notch2/NOTCH2* or *Jag1/JAG1* did not impair the myogenic ability of murine and human MABs *in vitro*. Furthermore, our *in vivo* data indicate that ligand-based tuning of the Notch pathway and its interactors may constitute a feasible integration-free strategy to potentiate the outcome of intra-arterial MAB therapy, possibly in combination with approaches to ameliorate microcirculation in dystrophic muscles.^24^ To potentially accelerate clinical translation of such approaches, it will be fundamental to MAB-specifically assess how the Notch pathway is involved in the interaction with other resident cell types and how it intertwines with other cascades, such as Wnt and Bone morphogenetic protein pathways.

## Materials and Methods

### Cell culture, isolation and differentiation

Murine AP^+^ MABs^12^ (*n*=3 clones from 3 individuals; p10-20) were cultured on collagen (Sigma-Aldrich, St. Louis, MO, USA)-coated plastic vessels (Nunc, Penfield, NY, USA) in DMEM20% medium (DMEM 4.5 g/l glucose, supplemented with 20% heat-inactivated fetal bovine serum, 1% Pen-Strep, 1% L-glutamine, 1% sodium pyruvate, 1% non-essential aminoacids, 0.2% 2-mercaptoethanol; all reagents by Life Technologies, Carlsbad, CA, USA) at 37 °C/5%O_2_. Human AP^+^ MABs (*n*=3 clones from 3 individuals; p8-15) were isolated by fluorescence-activated cell sorting (FACS) from human MAB populations^13^ by AP-PE antibody (R&D Systems, Minneapolis, MN, USA; 2 *μ*l/10^5^ cells) at FACS Aria III (BD, Franklin Lakes, NJ, USA). Human AP^+^ MABs were cultured on collagen (Sigma-Aldrich)-coated plastic vessels (Nunc) in IMDM10% medium (IMDM medium, supplemented with 10% heat-inactivated fetal bovine serum, 1% Pen-Strep, 1% L-glutamine, 1% sodium pyruvate, 1% non-essential aminoacids, 0.2% 2-mercaptoethanol, 5 ng/ml human FGF2 (Peprotech, Rocky Hill, NJ, USA)) at 37 °C/5%O_2_. Antigen profiling was performed with APC-conjugated antibodies (eBioscience, San Diego, CA, USA), following antibody specifications and appropriate isotypes, at FACS Canto (BD), then data were charted by FlowJo software (Tree Star, Ashland, OR, USA). AP enzymatic staining was performed incubating for 30 min at 37 °C 2% PFA-fixed cells with SigmaFast BCIP-NBT (Sigma-Aldrich) tablets dissolved in distilled water. Spontaneous differentiation was performed on collagen-coated vessels in DMEM2% medium (DMEM 4.5 g/l glucose, supplemented with 2% heat-inactivated horse serum, 1% Pen-Strep, 1% L-glutamine) at 37 °C/5%O_2_. C2C12 myoblasts (p5-12) were cultured in DMEM10% medium (DMEM 4.5 g/l glucose, supplemented with 10% heat-inactivated fetal bovine serum, 1% Pen-Strep, 1% L-glutamine, 1% sodium pyruvate) on plastic vessels (Corning, Corning, NY, USA) at 37 °C/5%O_2_. Differentiation in co-culture was performed seeding C2C12 myoblasts with MABs at 1 : 1 ratio, then applying DMEM2% medium after 24 h. GFP^+^ MABs were obtained by integration of a GFP-bearing transposon, as previously described.^12^

### WB, IP, qPCR and immunofluorescence/histological staining analyses

WB analyses were performed on 50 *μ*g starting protein extracts, previously obtained homogenizing cells or tissues in RIPA buffer supplemented with 1% PMSF 1 mM, 1% sodium fluoride 10 mM, 1% protease inhibitor cocktail, 1% sodium orthovanadate 0.5 mM (all reagents by Sigma-Aldrich). Gels were polymerized with 30% acrylamide (Sigma-Aldrich) according to assayed protein size (8% for MyHC; 10% for Dll1, NICD, Maml1; 12% for Gapdh, Mef2C, GFP) and electrophoresis/transfer/detection was performed on gels in parallel when assaying the same samples for multiple proteins. Incubation with primary antibody was performed in 5% skim milk TBS-T overnight at 4 °C, whereas HRP-conjugated secondary antibodies (1 : 5000, Santa Cruz, Dallas, TX, USA) were applied for 1 h at room temperature. Bands were revealed after 15 min incubation with SuperSignal Dura Chemiluminescence substrate (Thermo Scientific, Waltham, MA, USA) and acquired at GelDoc setup (Bio-Rad, Hercules, CA, USA). Quantitation was performed on gels loaded and blotted in parallel. Densitometric values of protein bands were quantitated through QuantityOne software (Bio-Rad), then normalized *versus* background noise and averaged values of the related Gapdh/GAPDH internal control bands. Primary antibodies, dilutions; mouse anti-MyHC (Developmental Studies Hybridoma Bank, Iowa, IA, USA; clone MF20), 1 : 10; rabbit anti-Dll1 (Cell Signaling, Beverly, MA, USA), 1 : 500; rabbit anti-Notch1 (Abcam, Cambridge, UK), 1 : 500; rabbit anti-NICD (Abcam), 1 : 500; rabbit anti-Maml1 (Millipore, Billerica, MA, USA), 1 : 500; rabbit anti-Gapdh (Sigma-Aldrich), 1 : 1000; rabbit anti-Mef2C (LifeSpan Biosciences, Providence, RI, USA), 1 : 500; rabbit anti-GFP (Life Technologies), 1 : 300.

IP was performed on 100 *μ*g protein extracts, previously obtained homogenizing cells in IP buffer (Tris-HCl pH 8 50 mM, NaCl 150 mM, 1% NP40, 0.5% sodium deoxycolate, 1% PMSF 1 mM, 1% sodium fluoride 10 mM, 1% protease inhibitor cocktail, 1% sodium orthovanadate 0.5 mM (all reagents by Sigma-Aldrich)). Immunoprecipitation was performed by overnight incubation with 0.5 *μ*g/reaction mouse anti-NICD (Abcam) in a total volume of 500 *μ*l IP buffer, while rotating at 4 °C. Bound fraction was precipitated by proteinG-sepharose beads (GE Healthcare, Cleveland, OH, USA) and separated from the unbound. Query proteins were then assayed on parallel membranes by rabbit primary antibodies.

For qPCR analyses, RNA was isolated through RNA mini kit, removing gDNA traces by Turbo DNase. One microgram RNA was reverse-transcribed by means of SuperScript III kit and qPCR was performed in 384-well plates (10 *μ*l final volume; thermal profile, 95 °C 15′′ −60 °C 45′′ ( × 50); ViiA 7 qPCR plate reader), using Platinum Sybr Green Mix, 1 *μ*l 1 : 5 diluted cDNA and 100 nM primers (all kits, reagents and plate reader by Life Technologies). Pgk/PGK was used as internal normalizer. The list of primers can be found in the Supplementary Materials and Methods.

Immunofluorescence staining was performed on PFA-fixed cells or cross-sectional 10 *μ*m-thick muscle slices, obtained by cryoembedding muscles in Tissue-Tek OCT (Sakura, Alphen aan den Rijn, The Netherlands) and cutting at CryoStar NX70 (Thermo Scientific) cryostat (−16 °C sample, −22 °C blade). Incubation with primary antibodies was performed in PBS supplemented with 5% BSA overnight at 4 °C, whereas AlexaFluor-conjugated secondary antibodies (1 : 500, Life Technologies) were applied for 1 h at room temperature. Nuclei were counterstained with 10 *μ*g/ml Hoechst (Sigma-Aldrich). Images were acquired at Eclipse Ti (Nikon, Tokyo, Japan) fluorescence inverted microscope, using NIS Elements AR 4.11.01 (Nikon) software with background ROI calibration optimized on isotype controls. Primary antibodies, dilutions; goat anti-GFP (Abcam), 1 : 500; rabbit anti-RFP (Bio-Connect, Huissen, The Netherlands), 1 : 200; mouse anti-MyHC (DSHB, clone MF20), 1 : 3; rabbit anti-laminin (Sigma-Aldrich), 1 : 300; mouse anti-Sgca (Leica Biosystems, Nussloch, Germany), 1 : 100; mouse anti-human DYSTROPHIN (Leica Biosystems, clone Dys3), 1 : 100; rabbit anti-human sarcomeric ACTININ*α* (Abcam), 1 : 250. Masson's trichromic staining was conducted on 5 *μ*m-thick cross-sectional paraffin-embedded muscle slices by means of staining kit (Sigma-Aldrich) according to manufacturer's instructions.

### DNA methylation assay

CpG islands were defined by submitting the 10 kb gDNA sequence upstream of the transcription start to CpG island searcher (http://cpgislands.usc.edu; lower limits, CpG%=55, ObsCpG/ExpCpG=0.65, length=500, distance=100). CpG island in murine Dll1 locus, −4257 to −3258 bp from transcription start. CpG islands in human DLL1 locus, −5909 to −3112 bp and −503 to −0 bp from transcription start. gDNA was isolated from 10^6^ cells in proliferative conditions by means of genomic DNA Mini kit (Life Technologies) and 1 *μ*g gDNA was randomly fragmented into fragments of 200–300 bp in 50 *μ*l TE buffer in Bioruptor sonication bath (Diagenode, Liège, Belgium) at 4 °C for 15 cycles (30′′ sonication/30′′ rest) at high intensity. Sheared gDNA (200 ng) was then enriched for fragments containing ≥5 me-CpGs using MethylCollector Ultra kit (Active Motif, Carlsbad, CA, USA; low salt conditions) and purified via MinElute Reaction CleanUp kit (Qiagen, Venlo, The Netherlands). Purified DNA was then assayed for specific amplification of sequential fragments of regulatory regions through Sybr green-based qPCR, using *APC* promoter (negative control, unmethylated) and *NBR2* promoter (positive control, methylated) as standards for relative quantification of the methylation propensity of the single fragments. The list of primers can be found in the Supplementary Materials and Methods.

### Knockdown, overexpression and luciferase reporter vectors

For knockdown of *Dll1/DLL1* and *Notch1/NOTCH1*, specific shRNA-mimicking oligonucleotides were PCR-amplified and cloned at the 3′ of the fluorescent tracer into pGIPZ (efficient in murine MABs; reporter, GFP) or pTRIPZ (efficient in human MABs; reporter, RFP) backbones (Open Biosystems, Huntsville, AL, USA). Vectors carrying the scramble shRNA control were purchased. Knockdown vectors were then used to produce lentiviral particles in 293T cells, using 2nd generation packaging plasmids. At 24 h after application of the viral supernatant, transduced MABs were sorted for the tracer and then plated for knockdown efficiency check and for differentiation. MABs pre-treated for 48 h with medium supplemented with 1 : 500 *γ*-secretase inhibitor X (Millipore) were also then incorporated in the differentiation experiment as control of chemical inhibition of the signaling. The list of oligonucleotides can be found in the Supplementary Materials and Methods.

For doxycycline-driven overexpression of *Dll1/DLL1* and *Mef2C/MEF2C*, full-length cDNAs were purchased as in between recombining sequences compatible for Gateway system (Genecopoeia, Rockville, MD, USA) or cloned into pENTR-11 shuttle vector (Life Technologies) from non-compatible plasmids (Open Biosystems). cDNA sequences were then recombined into pLOVE lentiviral backbone using LR Clonase II kit (Life Technologies), according to manufacturer's instructions. Lentiviral particles were produced in 293T cells, using 3rd generation packaging plasmids (5 ml viral suspension/75 cm^2^ 293T cells). Two milliliters of viral suspension were used to transduce 5 × 10^5^ cells. Twenty-four hours after transduction, doxycycline was added for 72 h according to the different concentrations, and then removed during the second part of differentiation. In case of gsi-treated control, gsi was added in combination with the doxycycline. Doxycycline-driven overexpression of *Dll1/DLL1* and *Mef2C/MEF2C* has been used for all *in vitro* experiments reported in Figures 1,2,3 and related Supplementary Figures.

Luciferase activity was monitored by transfecting transduced cells with RSRF-luc-2wt^25^ (Addgene, Cambridge, MA, USA) and renilla luciferase plasmids by means of Effectene (Qiagen). Luciferase activity was quantified at day 5 of *in vitro* spontaneous differentiation, normalizing firefly luciferase activity to renilla luciferase read per sample (Dual Reporter Luciferase assay kit by Promega, Madison, WI, USA) at EG&G microplate luminometer LB96V (Berthold, Wildbad, Germany).

For the adenoviral-based strategy, cDNAs in pENTR-11 shuttle vector were recombined, as mentioned before, into pAd-CMV-V5-DEST, whereas pAd-CMV-V5-GW-*lacZ* was used as Ad-mock control (both vectors by Life Technologies). Adenoviral particles were produced by transfecting PacI-linearized pAd vectors into 25 cm^2^ 293A cells (final volume=2 ml). Upon 100% mortality, I supernatant was used to transduce 150 cm^2^ 293A cells (final volume=10 ml). Upon 100% mortality, II supernatant was frozen in 2-ml aliquots and stored at −80 °C. One vial was used to transduce 5 × 10^5^ proliferating MABs and, after 48 h, cells were carefully washed, collected and resuspended in the appropriate number/volume conditions for injection.

### *In vivo* experiments and evaluation of the functional outcome

All animal protocols were conducted in compliance with Ethical Committee Guidelines of KU Leuven (project 095/2012) and Belgian legislation. *Sgca-null* (C57/Bl6 background) dystrophic mice (6-months old) were generated by the group of Prof. K.P. Campbell (University of Iowa, IA, USA).^26^ *Rag2-null/γc-null* immunodeficient mice (2-months old) were provided by the group of C. Verfaillie (KU Leuven, Belgium). Animals were anesthetized with isofluorane. Bilateral intra-femoral artery injection was performed with 2.5 × 10^5^ cells/50 *μ*l saline solution supplemented with 1 : 10000 heparin (LEO Pharma, Ballerup, Denmark)/femoral artery, using 32-gauge needles under STEMI SV11 stereomicroscope (Zeiss, Oberkochen, Germany). Mice were kept under cyclosporine (Sandimmune Cyclosporine, Novartis, Basel, Switzerland; 10 mg/kg) regimen during the whole treatment. Functional outcome was measured through gait analysis^27^ and treadmill assay.^28^ Both assays were first tested and validated comparing age-matched, background-matched mice to dystrophic mice (data not shown). Gait analysis was performed inking the back paws and letting the mice freely walk along a 1-m-long paper ribbon, confined into a walled plastic path. More than three runs per time point and ≥25 stride length measurements were analyzed per mouse/time point. Treadmill assay was performed on 10° uphill-oriented treadmill belt, with 10 m/min starting speed and 1 m/min^2^ acceleration. Mice were stopped after ≥5 consecutive seconds on the pulse grills.

### Flx mice derivation, flx cell isolation and conditional Dll1 knockout

*Dll1*^*flx/+*^ mice were rederived from frozen 2-cell stage embryos, obtained from Prof. Julian Lewis (Cancer Research UK),^29^ crossed with *Rosa26::iCreERT2*^*+/−*^ mice, obtained from Prof. Massimiliano Mazzone (VIB, KU Leuven, Belgium), backcrossed on C57/Bl6 background until obtaining *Dll1*^*flx/+*^*;Rosa26::iCreERT2*^*+/−*^ (flx) mice. Flx MABs were isolated from the skeletal muscle following the procedures used for wild-type murine MABs, as previously reported.^12,14^ Flx SCs were isolated by digesting hind limb skeletal muscles of newborn mice in PBS supplemented with 0,04% Collagenase V/0,06% Pancreatin (Sigma-Aldrich) while shaking at 37 °C. After filtration and enzyme blockade, the cell suspension was FACS-purified by means of SMC2.6 antibody, provided by Prof. So-ichiro Fukada (University of Osaka, Japan). Flx SCs were cultured on collagen-coated Nunc plastic with DMEM20% medium supplemented with 1% chicken embryo extract (Bio-Connect). Intra-arterial injections were performed as mentioned above.

Conditional *Dll1* knockout was performed by means of seven intra-peritoneal injections (every second day) of 3 mg tamoxifen dissolved in 50 *μ*l corn oil (both reagents by Sigma-Aldrich) into 1-month-old flx females; allele removal was checked by PCR on 1 ng gDNA 5 days after last injection (primers, Fw 5′-accttctttcgcgtatgcctcaag-3′, Rev, 5′-agagtctgtatggagggcttc-3′). Conditional *Dll1* knockout in cells was performed by adding 10 *μ*M 4-OH-tamoxifen (Sigma-Aldrich), dissolved in ethanol, to the growth medium for 5 days consecutively. Once PCR-checked for allele removal, *Dll1*-knockout cells were then used for *in vitro* differentiation or *in vivo* injection.

### Statistical analysis

Sample size for *in vitro/in vivo* experiments was calculated by means of Sample Size Calculator (http://www.stat.ubc.ca/~rollin/stats/ssize/index.html; parameters: power,.80; alpha,.05). When applicable, sample size analysis was based on average values obtained from preliminary optimization/validation trials. To analyze data pools from methylation propensity and qPCR assays, one-way ANOVA (to test difference among >2 pools) and unpaired *t*-test (to compare two specific pools) were used. Significance was achieved when *P*<0.05 in both tests. To analyze data pools of luciferase activity, protein levels, functional outcome, fiber count, fusion index and Pax7^+^ nuclei count assays, Kruskal–Wallis and Mann–Whitney *U* test were used. Significance was accepted when *P*<0.05 was scored in both tests. All statistical tests were performed by means of Prism software (GraphPad, La Jolla, CA, USA).

## Abbreviations

Ad: adenoviral vector
AP: Alkaline phosphatase
AU: arbitrary units
cm: centimeters
CpG: cytosine guanine (linear dinucleotide)
Dll1: Delta-like ligand 1
hDYS: human-specific DYSTROPHIN
EudraCT: European Union Drug Regulating Authorities Clinical Trials
flx: floxed, that is, comprised between two loxP sites
Gapdh: Glyceraldehyde-3-phosphate dehydrogenase
GFP: Green fluorescent protein
gsi: γ-secretase inhibitor
Hes1: Hes family bHLH transcription factor 1
HLA: human leukocyte antigen
iCreERT2: inducible Cre recombinase fused with estrogen receptor (tamoxifen-responsive) 2
IgG: Immunoglobulin G
IP: immunoprecipitation
Jag1: Jagged 1
lucif: firefly luciferase
m: meters
MABs: mesoangioblasts
Maml1: Mastermind-like 1
MDs: muscular dystrophies
Mef2C: Myocyte enhancer factor 2C
min: minutes
MyHC: Myosin heavy chain
NICD: Notch1 intracellular domain
Pdgf-b: Platelet derived growth factor receptor, beta polypeptide
Pgk: Phosphoglycerate kinase
qPCR: quantitative polymerase chain reaction
Rag2: Recombination activating gene 2
RFP: Red fluorescent protein
Sgca: Sarcoglycan α
WB: western blot
Wnt: wingless-type MMTV integration site family
γc: interleukin 2 receptor, gamma chain
