# Supplementary figures and images for: Notch signaling regulates myogenic regenerative capacity of murine and human mesoangioblasts
Source: Cell Death Dis. 2014 Oct 9;5(10):e1448–. doi: 10.1038/cddis.2014.401 (PMC4237240; doi:10.1038/cddis.2014.401)

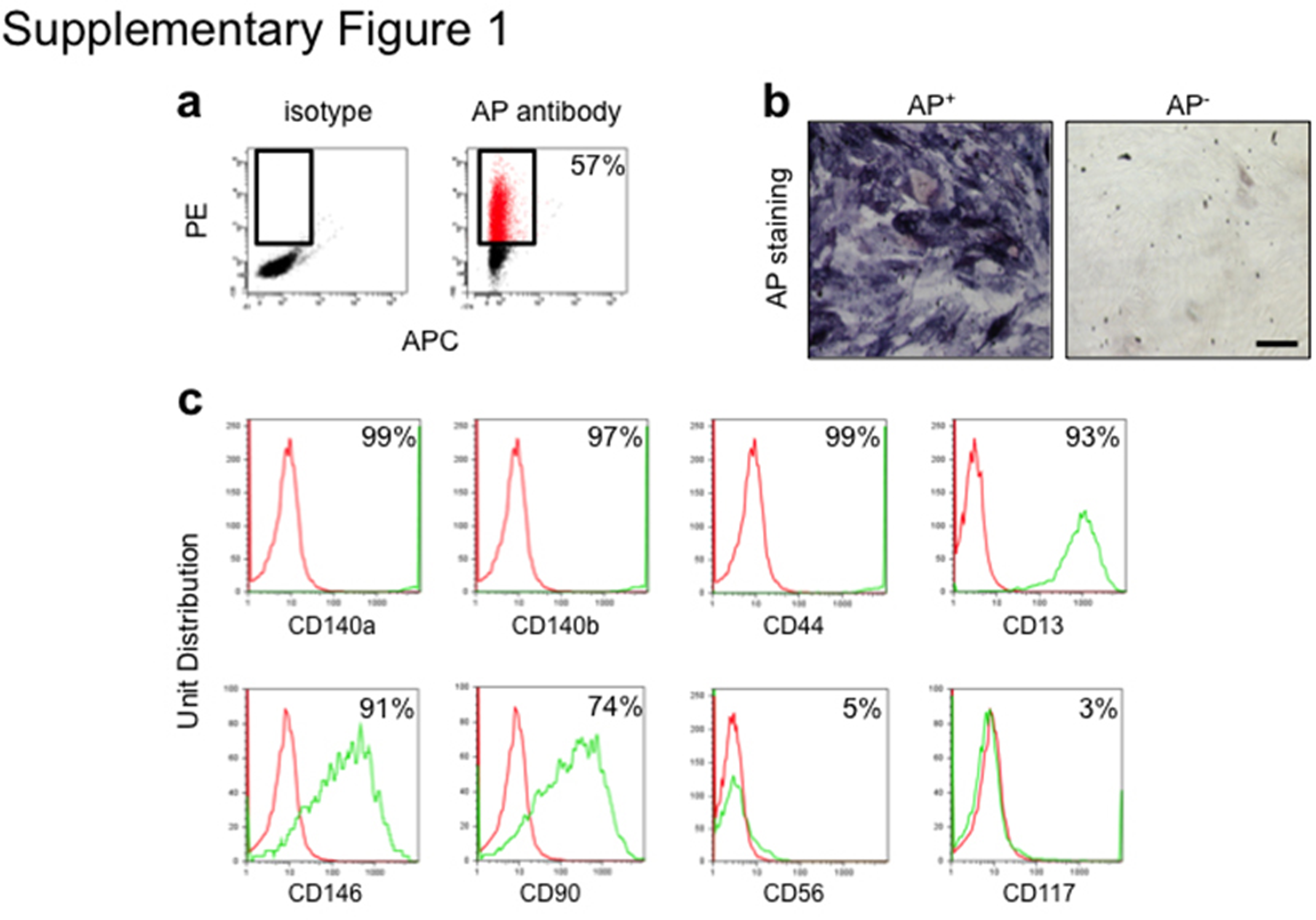

Supplement: Supplementary Figure 1 [file cddis2014401x1.tif]

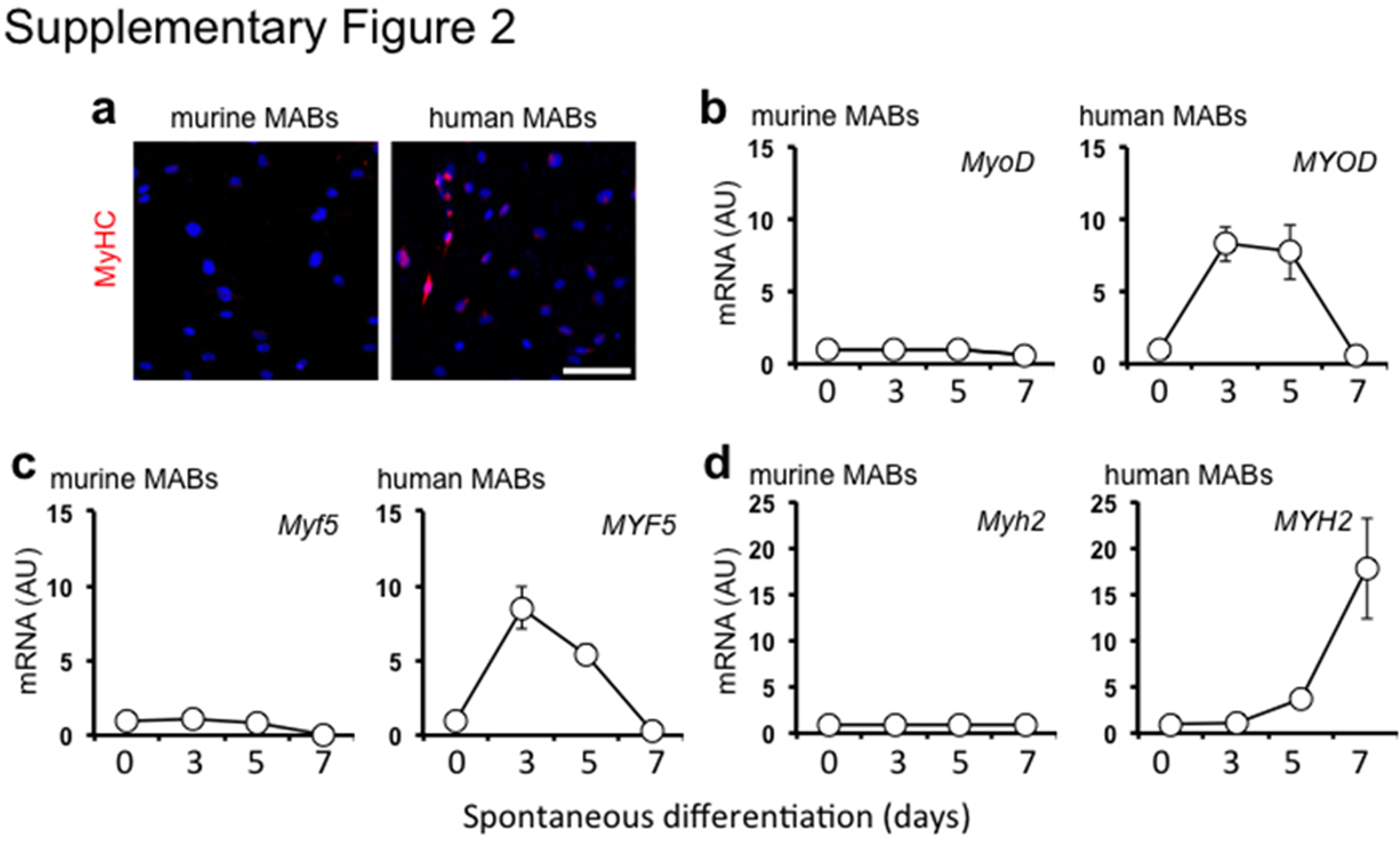

Supplement: Supplementary Figure 2 [file cddis2014401x2.tif]

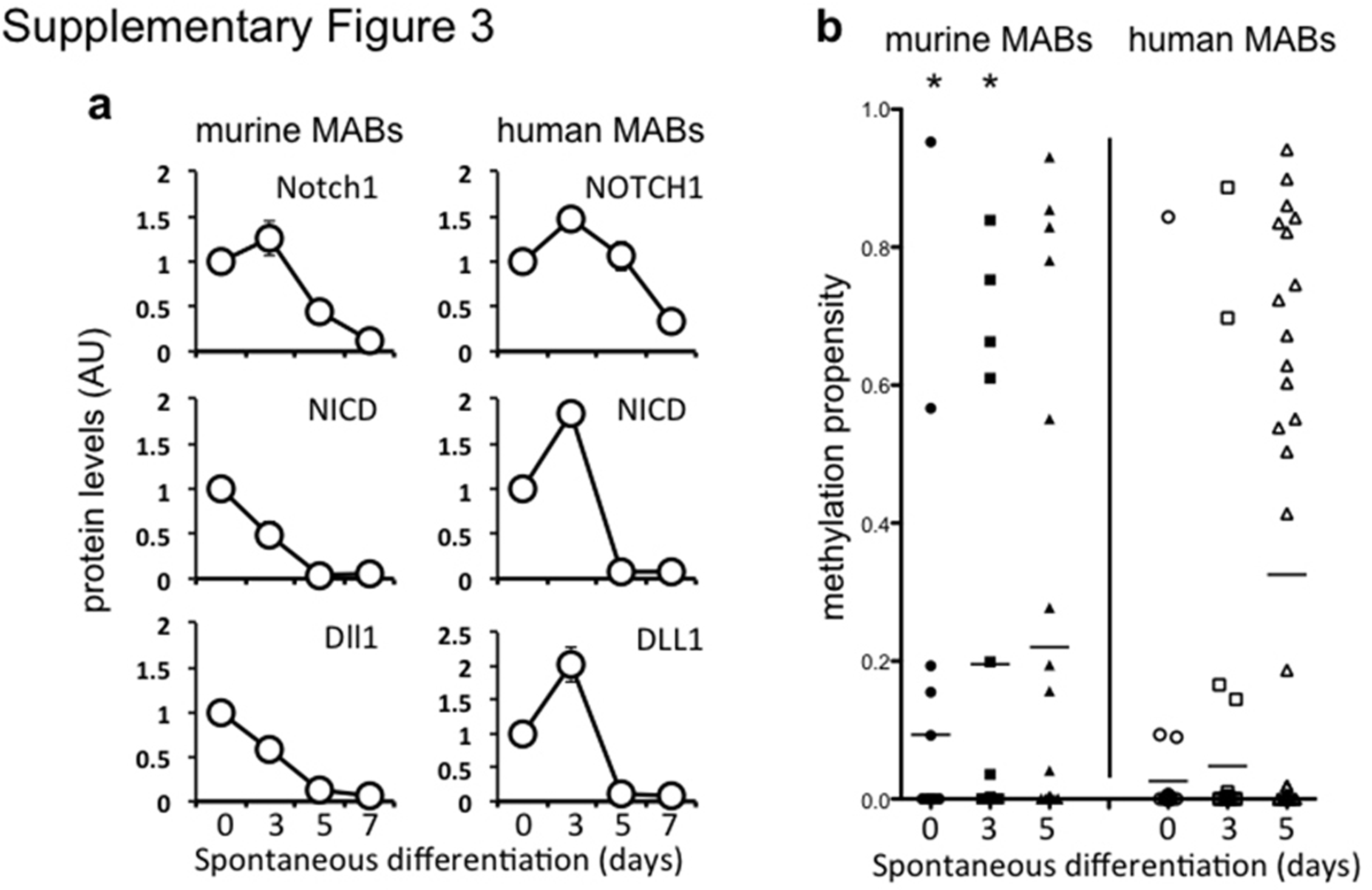

Supplement: Supplementary Figure 3 [file cddis2014401x3.tif]

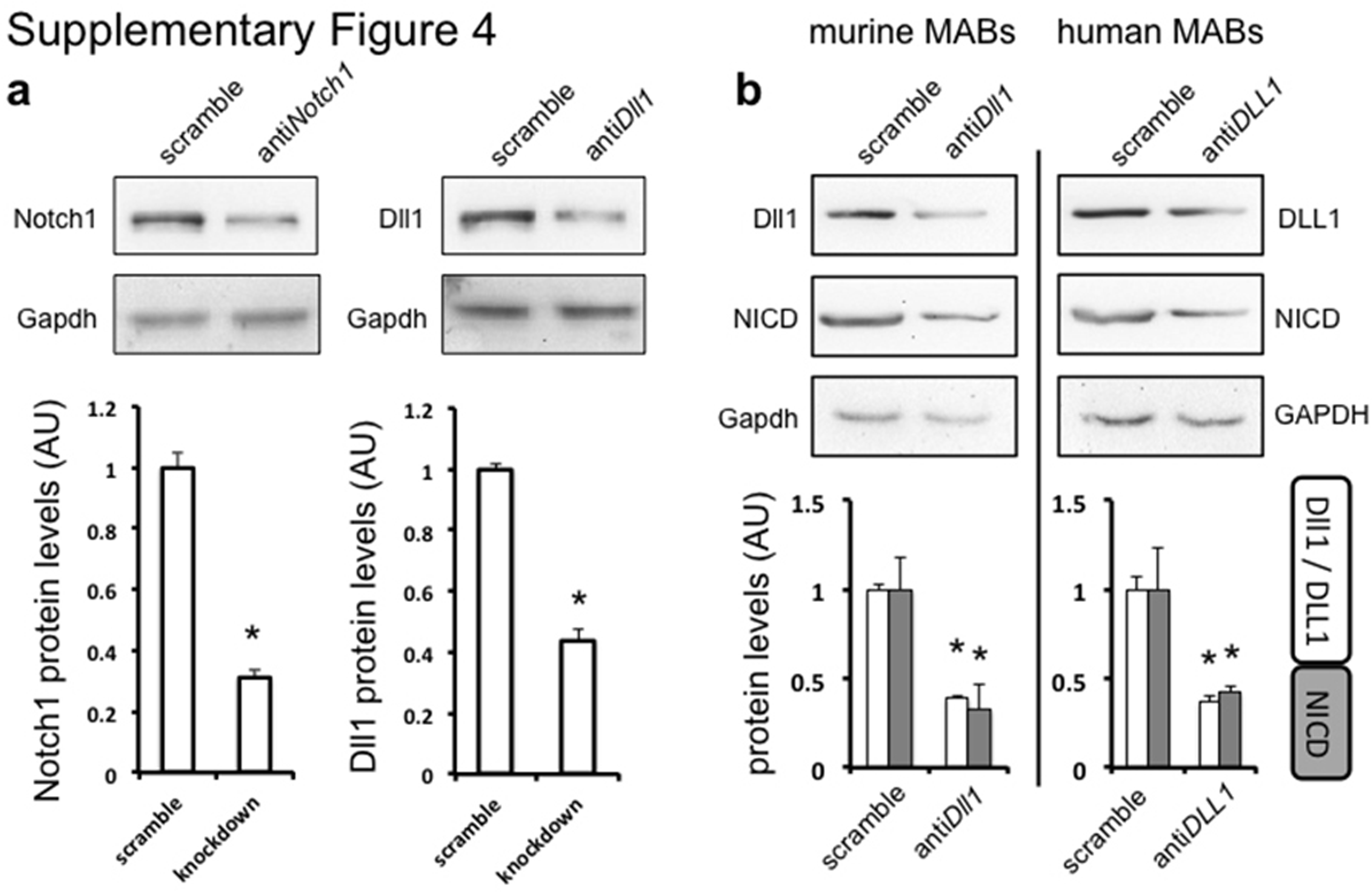

Supplement: Supplementary Figure 4 [file cddis2014401x4.tif]

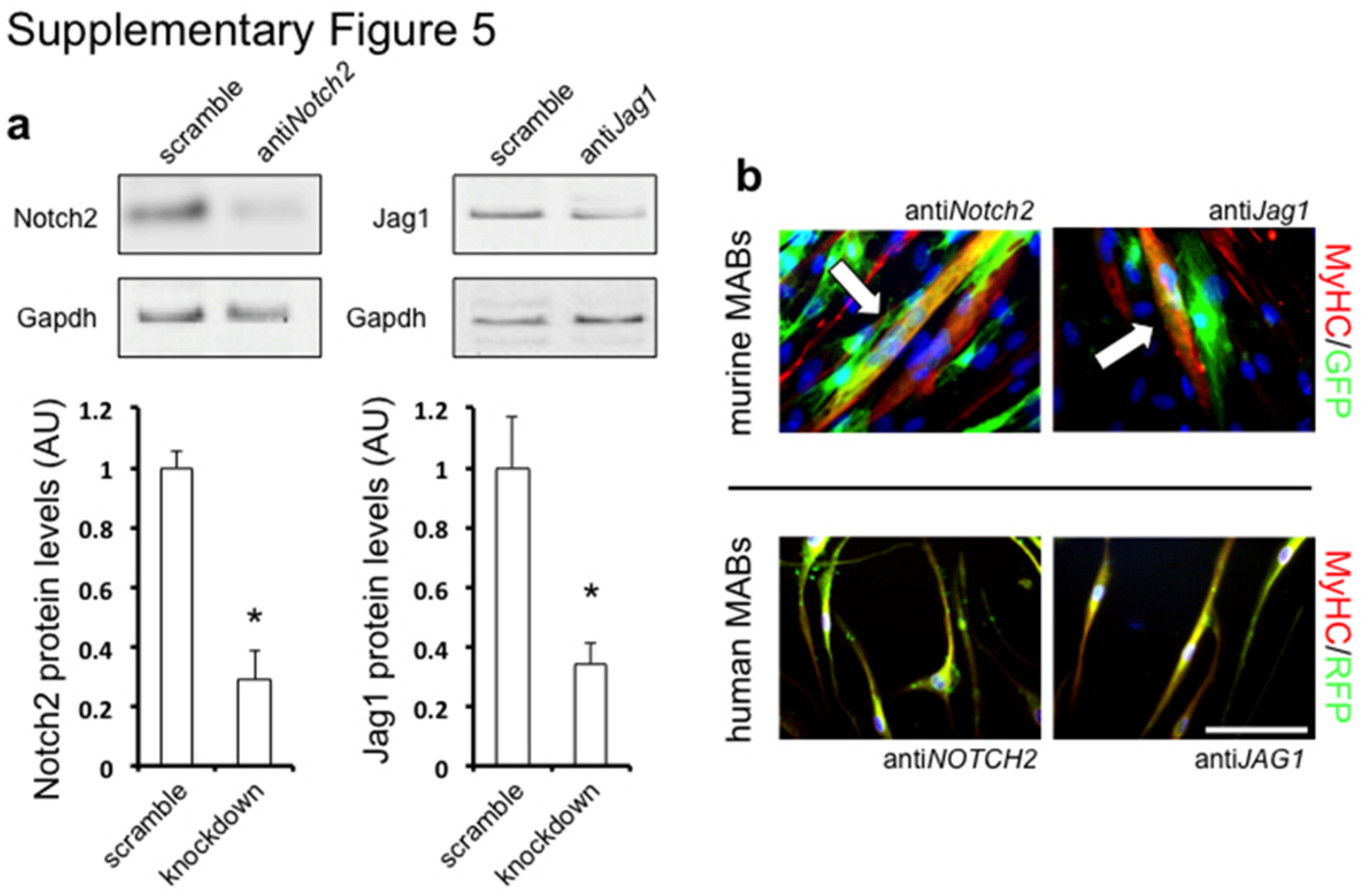

Supplement: Supplementary Figure 5 [file cddis2014401x5.tif]

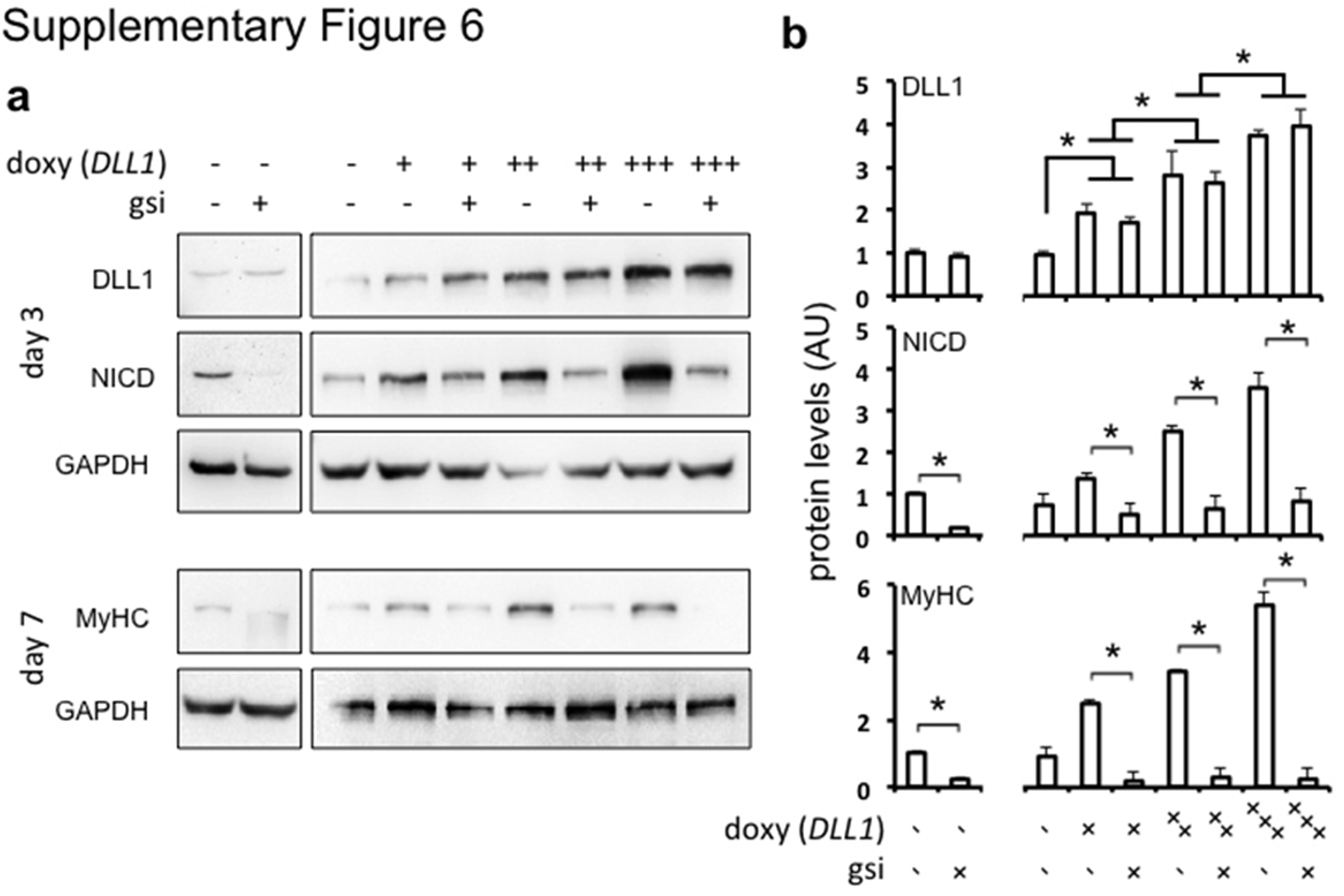

Supplement: Supplementary Figure 6 [file cddis2014401x6.tif]

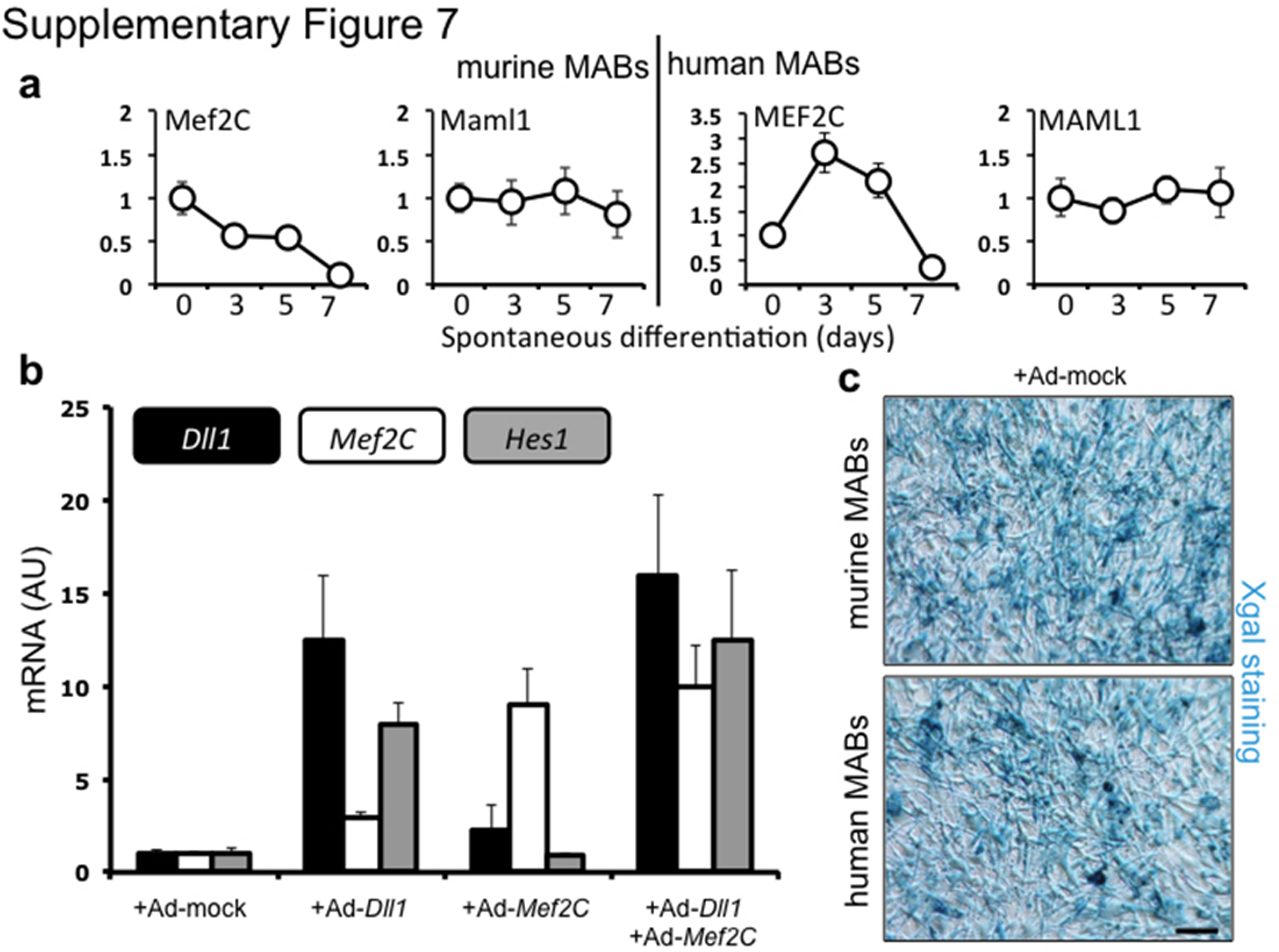

Supplement: Supplementary Figure 7 [file cddis2014401x7.tif]

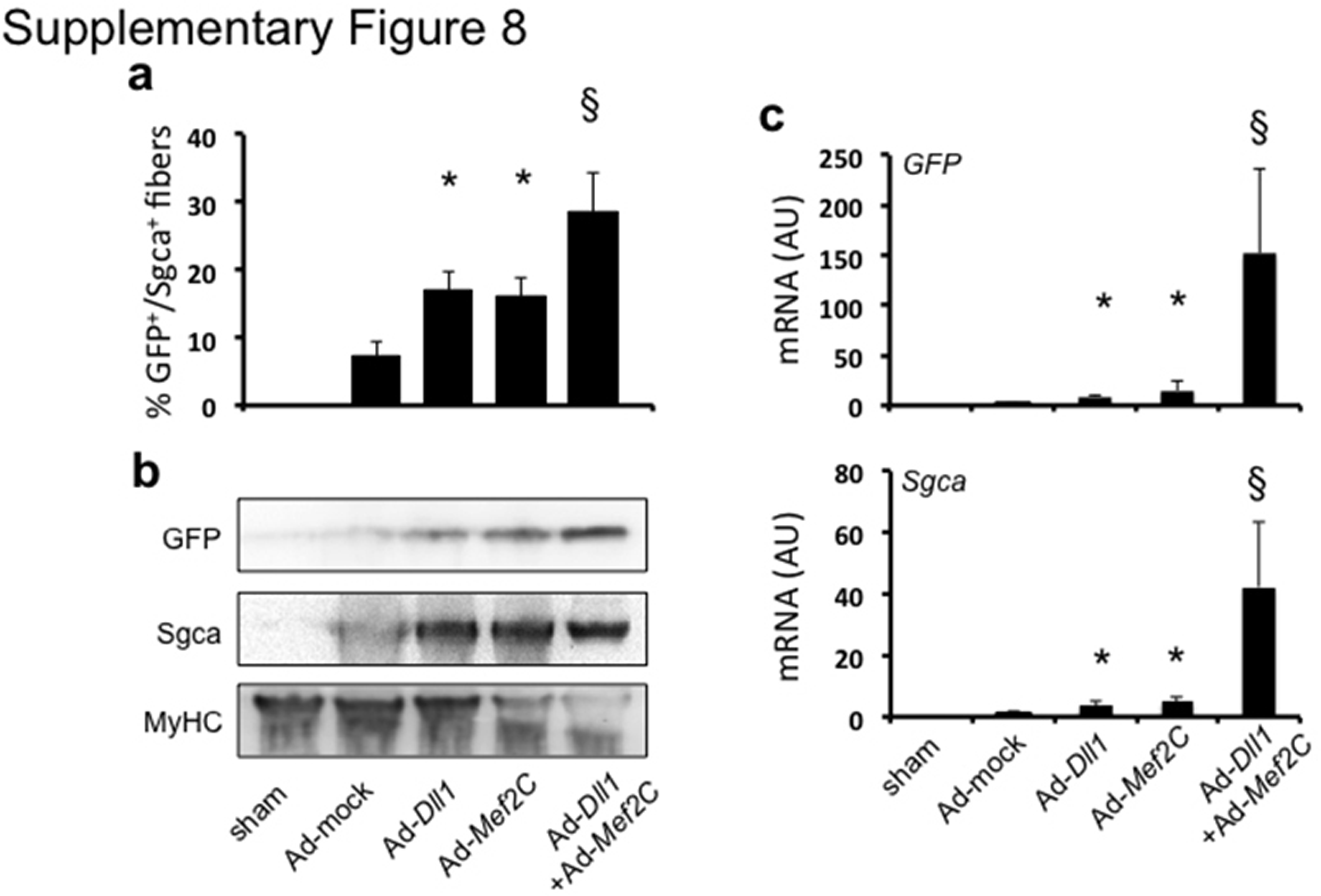

Supplement: Supplementary Figure 8 [file cddis2014401x8.tif]

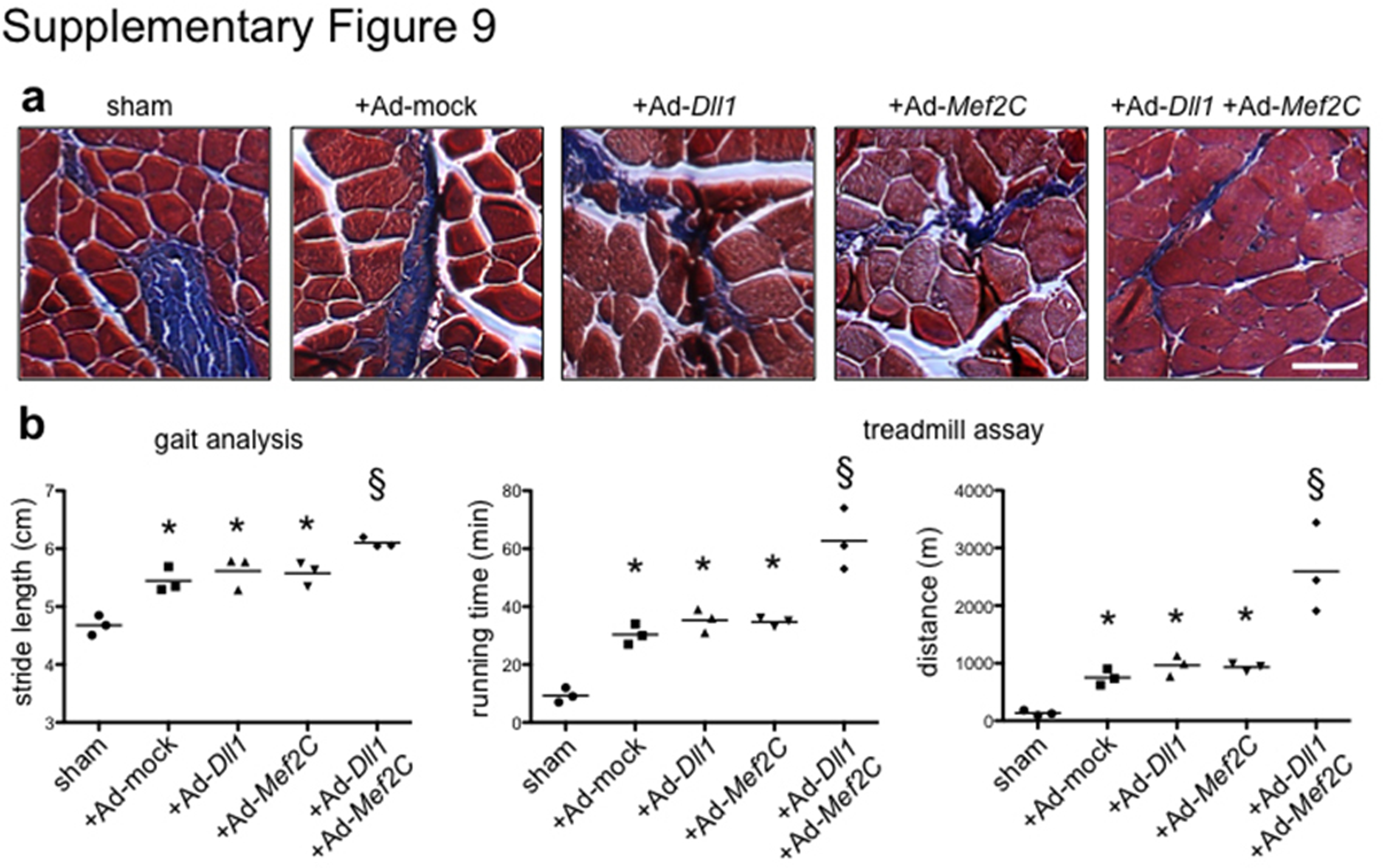

Supplement: Supplementary Figure 9 [file cddis2014401x9.tif]

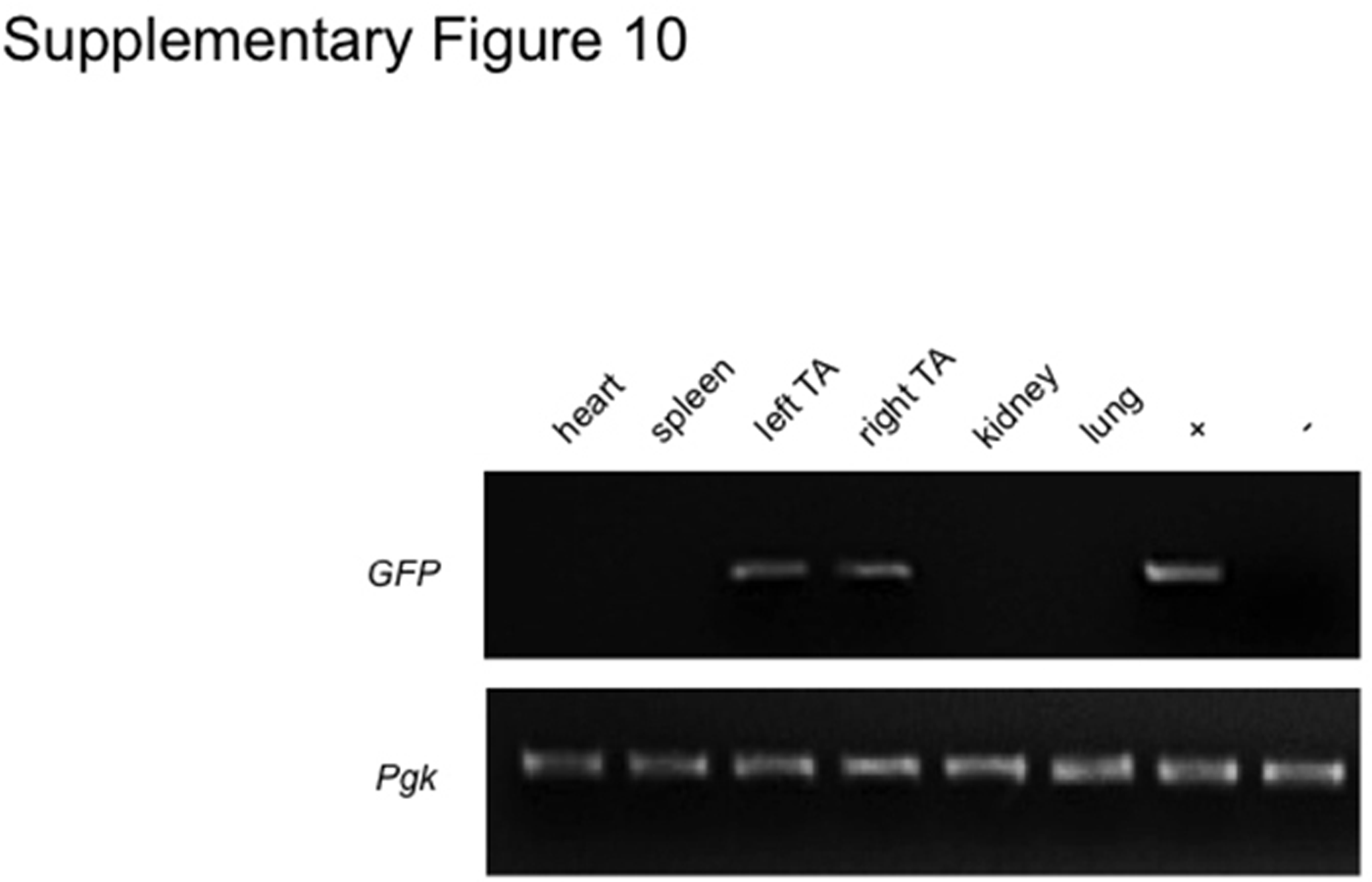

Supplement: Supplementary Figure 10 [file cddis2014401x10.tif]

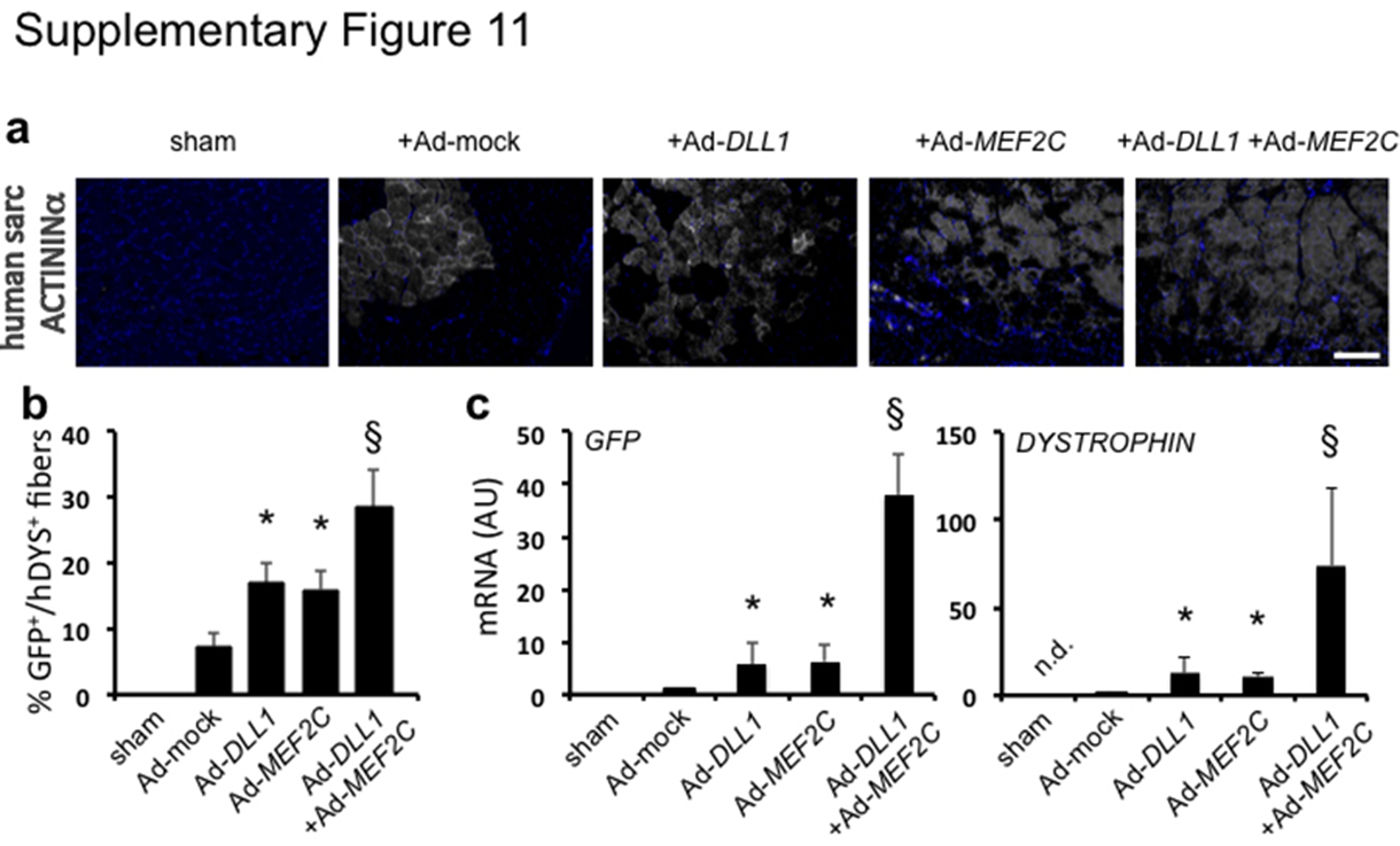

Supplement: Supplementary Figure 11 [file cddis2014401x11.tif]

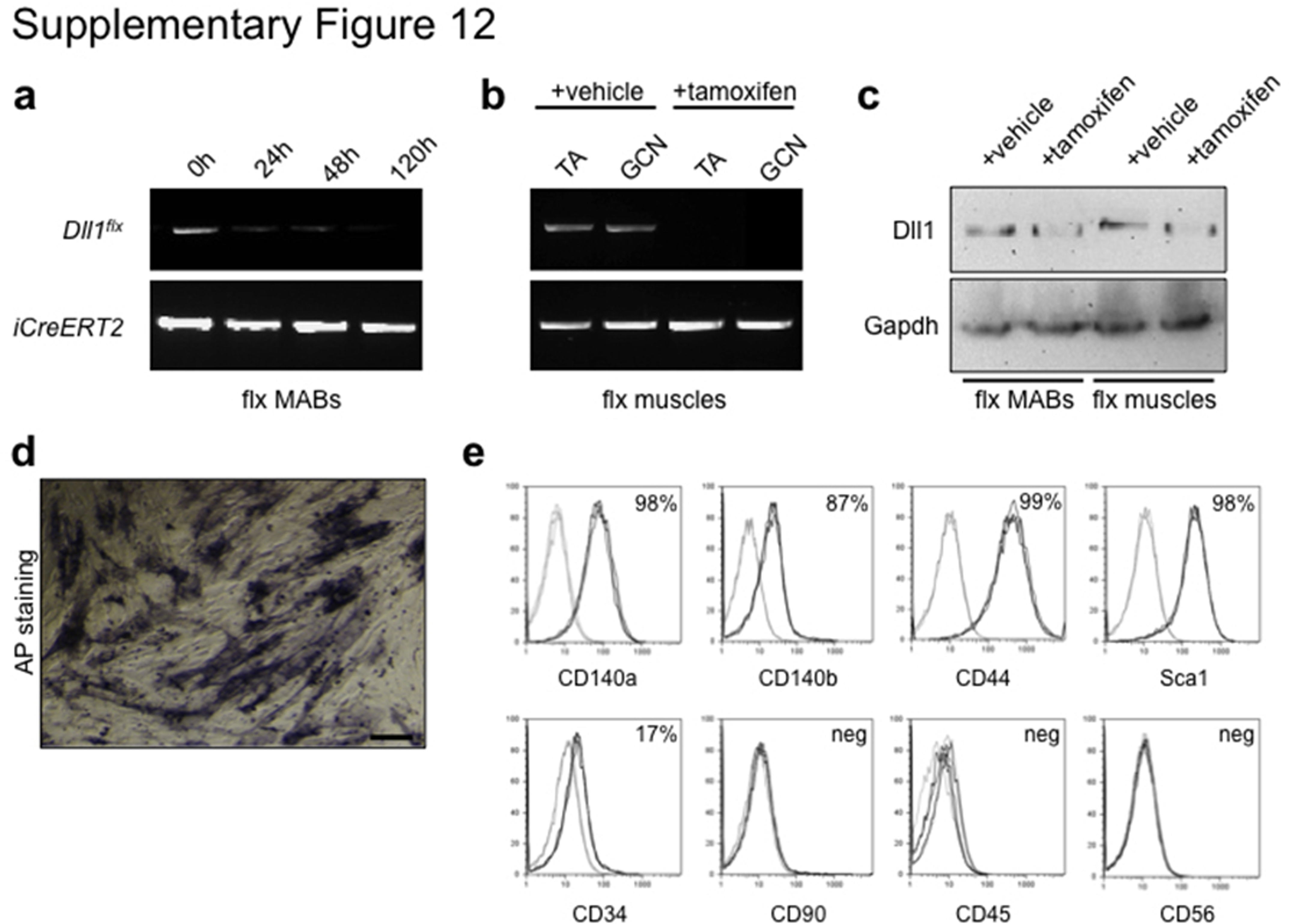

Supplement: Supplementary Figure 12 [file cddis2014401x12.tif]

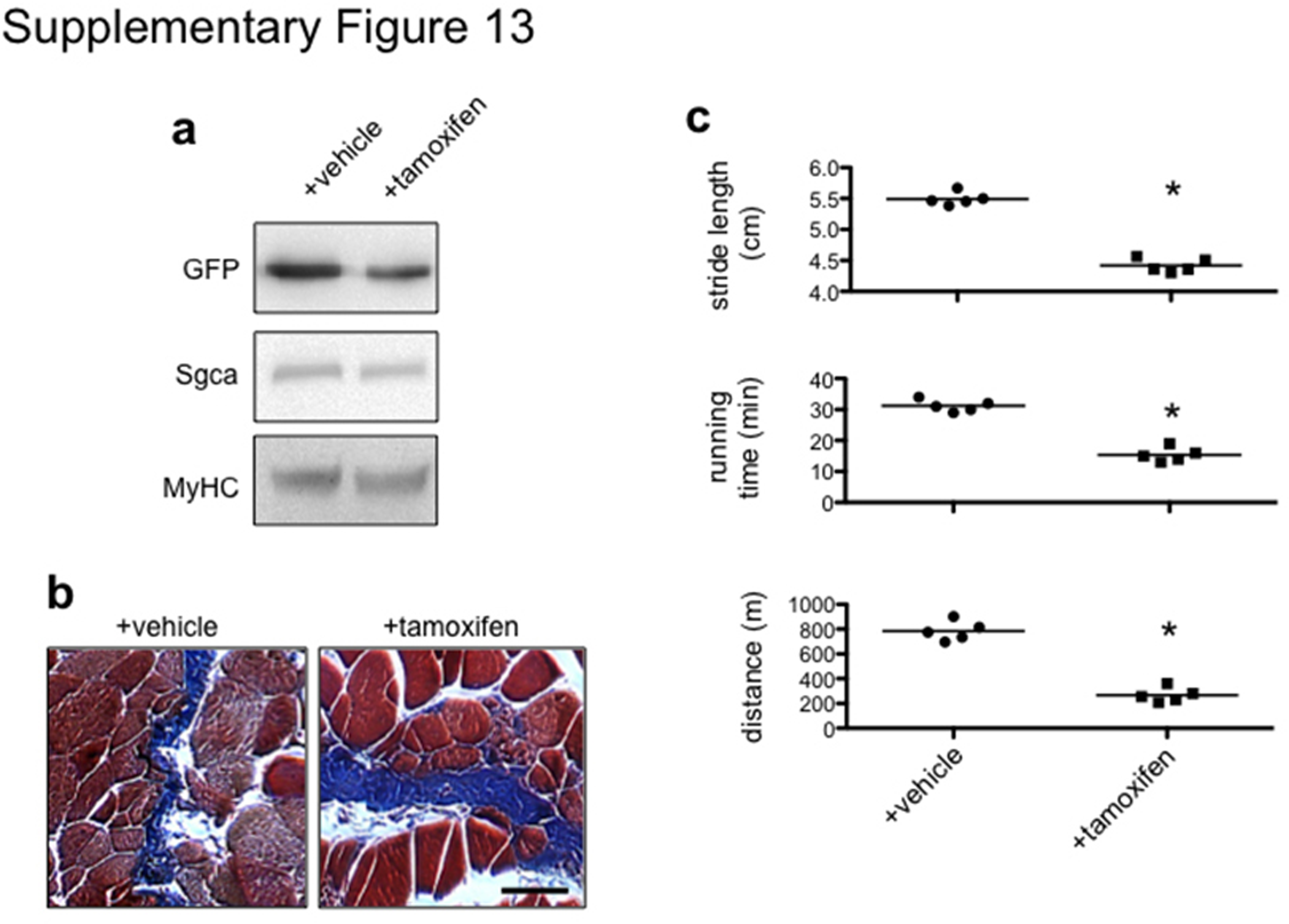

Supplement: Supplementary Figure 13 [file cddis2014401x13.tif]

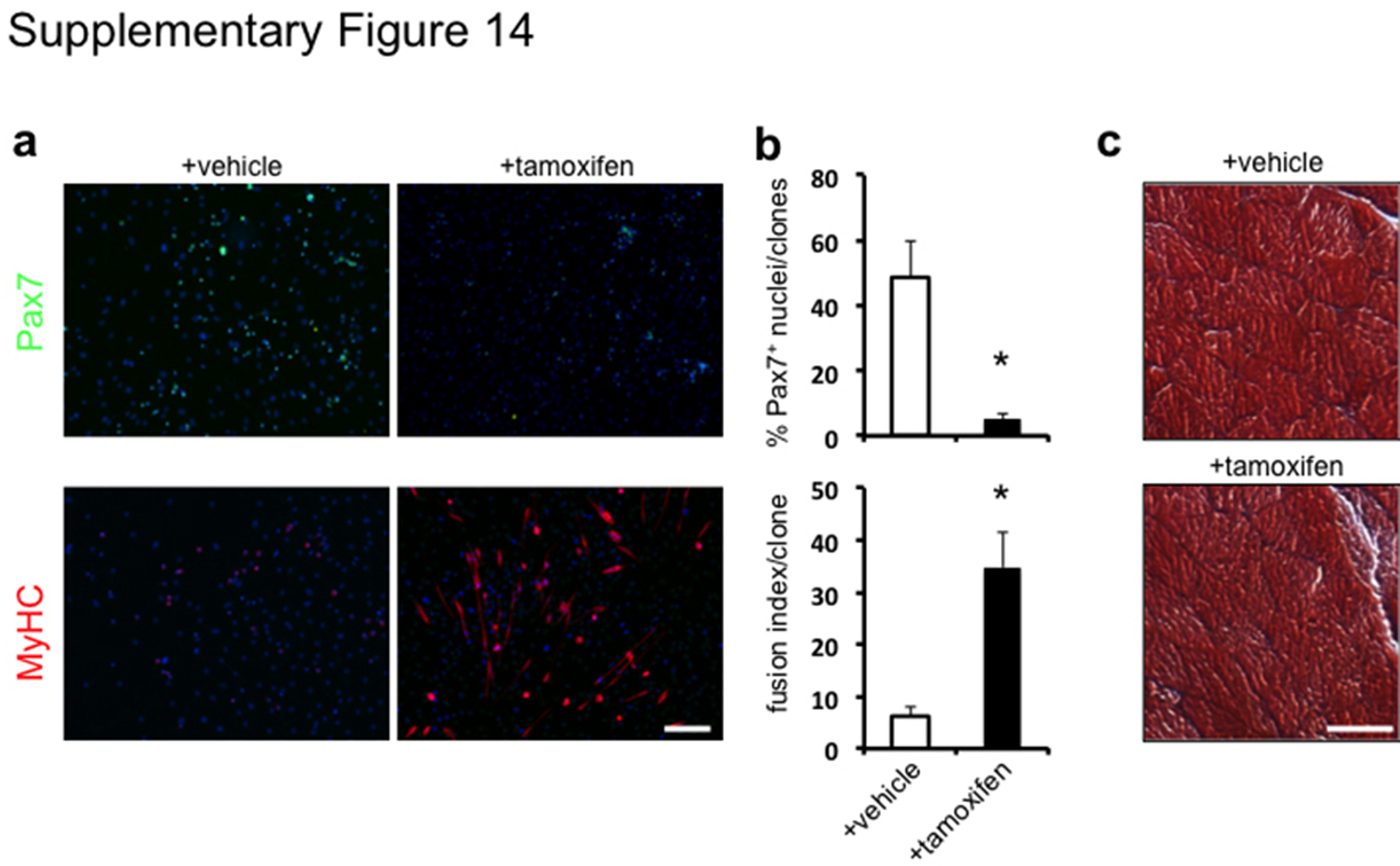

Supplement: Supplementary Figure 14 [file cddis2014401x14.tif]
