# Supplementary material for: Notch signaling regulates myogenic regenerative capacity of murine and human mesoangioblasts
Source: Cell Death Dis. 2014 Oct 9;5(10):e1448–. doi: 10.1038/cddis.2014.401 (PMC4237240; doi:10.1038/cddis.2014.401)
Supplement: Supplementary Figure Legends [file cddis2014401x15.doc]

**List of Supplementary Figure Legends**

**Supplementary Figure 1. FACS isolation and characterization of AP^+^ human MABs.** In order to analyze human MABs with the same prospective isolation method as the murine MABs, human MAB clones (previously reported ^13^) were sorted for AP (**a**, lined gate indicates AP^+^ population) and confirmed for AP expression by colorimetric enzymatic test, as compared to AP^-^ population **(b)**. **(c)** AP^+^ human MABs display the typical antigen profile for these cells under proliferative conditions ^14^; red line, isotype; green line, AP^+^ human MABs. Scale bar indicates 100μm.

**Supplementary Figure 2.** **Comparison of *in vitro* spontaneous myogenic capacity between murine and human MABs. (a)** At 7 days of spontaneous differentiation, murine MABs fail in differentiating into MyHC^+^ myocytes, unlike human MABs. **(b-d)** Human MABs show transient activation of myogenic transcription factors *MYOD* and *MYF5* at day 3-5 and of the late marker *MYH2* at day 5-7, unlike murine MABs. Expression levels are reported in arbitrary units (AU) as fold change vs day 0. Data in charts are depicted as mean ± standard deviation of ≥3 independent experiments. Scale bar indicates 100μm.

**Supplementary Figure 3. Quantitation of protein levels and Dll1/DLL1 locus methylation during spontaneous differentiation.** **(a)** Although the protein levels of Notch1/NOTCH1 receptor follow a similar pattern, NICD levels parallel the trend of Dll1/DLL1. Protein levels are reported in arbitrary units (AU) as fold change vs day 0. Data in charts are depicted as mean ± standard deviation of 3 independent experiments. **(b)** Depicted in the chart are the single average propensity values (represented as 0-to-1 values, normalized vs positive control) of each assayed fragment along the regulatory regions at day 0-3-5 during spontaneous differentiation. Considering all the fragments together, murine regulatory regions appear significantly more methylated at day 0 and 3; *, P<0.05 vs human, n=3.

**Supplementary Figure 4.** **Analysis of Notch1/NOTCH1 and Dll1/DLL1 knockdown at protein levels in murine and human MABs.** **(a)** At 48hours after transduction with shRNA-bearing vectors, both genes appear significantly downregulated even in protein levels, as compared to cells transduced with scramble shRNA-bearing vector. Depicted are analyses on murine MABs, human MABs presented analogous data (data not shown). (b) In both murine and human MABs, ligand knockdown resulted in significant decrease of NICD levels at 48hours after transduction. Data in charts are depicted as mean ± standard deviation. Protein levels are reported in arbitrary units (AU) as fold change vs scramble control; *, P<0/05, n=3.

**Supplementary Figure 5.** **Knockdown of Notch2/NOTCH2 and Jag1/JAG1 does not alter MAB differentiation. (a)** Protein level analyses at 48hours post-transduction. Depicted are analyses on murine MABs, human MABs presented analogous data (data not shown). **(b)** In both murine and human MABs, knockdown of Notch2/NOTCH2 and Jag1/JAG1 did not affect the myogenic capability *in vitro*, unlike Notch1/NOTCH1 and Dll1/DLL1 (compare to **Figure 1g-h**). Data in charts are depicted as mean ± standard deviation. Protein levels are reported in arbitrary units (AU) as fold change vs scramble control; *, P<0/05, n=3. Scale bar indicates 100μm.

**Supplementary Figure 6.** ***DLL1* dose-dependently enhances *in vitro* myogenic ability of human MABs. (a)** WB analysis and **(b)** protein level quantitation of differentiating human MABs after transient (day 0-3) doxycycline-mediated overexpression of the ligand. DLL1 protein levels appear dose-dependently significantly upregulated, and NICD levels accordingly increased at day 3. MyHC resulted accordingly upregulated at day 7, endpoint of the differentiation. Interestingly, when combined with gsi, *DLL1* overexpression significantly fails in increasing NICD and MyHC levels. Data in charts are depicted as mean ± standard deviation. Protein levels are reported in arbitrary units (AU) as fold change vs doxy-/gsi- control; *, P<0/05, n=3.

**Supplementary Figure 7.** **Priming murine MABs with adenoviral vectors promoting overexpression of *Dll1* and *Mef2C*.** **(a)** Quantitation of Mef2C/MEF2C and Maml1/MAML1 protein levels in murine and human MABs in conditions of spontaneous differentiation. Protein levels are reported in arbitrary units (AU) as fold change vs day 0. Data in charts are depicted as mean ± standard deviation of 3 independent experiments. **(b)** At 48hours after transduction *in vitro*, Ad-*Dll1* induces overexpression of *Dll1* and increased levels of *Hes1*, whereas Ad-*Mef2C* prevalently induces *Mef2C* overexpression. Combined priming with Ad-*Dll1* and Ad-*Mef2C* results in increased levels of both genes and of *Hes1*. Expression levels are reported in arbitrary units (AU) as fold change vs MABs transduced with Ad-lacZ (Ad-mock). Data in charts are depicted as mean ± standard deviation of ≥3 independent experiments. **(c)** In our conditions of virus production and transduction, kept constant for all adenoviral vectors, exposure of MABs to Ad-mock results in virtually complete transduction, as assayed by X-Gal staining at 48hours post-transduction. Scale bar indicates 100μm.

**Supplementary Figure 8.** **Beneficial effects of combined priming of murine MABs with *Dll1* and *Mef2C* on *in vivo* engraftment and regeneration.** **(a)** At 4 weeks after intra-arterial injection, priming of GFP^+^ murine MABs with either Ad-*Dll1* or Ad-*Mef2C* results in higher rates of GFP^+^/Sgca^+^ fibers in *gastrocnemius* muscles, as assayed by immunofluorescence staining. Moreover, combined priming with both vectors results in higher rates of engrafted and regenerated fibers, as compared to single priming. This is confirmed by WB **(b)** and qPCR **(c)** analyses on *tibialis anterior* muscles. Expression levels are reported in arbitrary units (AU) as fold change vs Ad-mock. *, P<0.05 vs sham; §, P<0.05 vs Ad*-Dll1* and vs Ad-*Mef2C* (n=3 mice/group) Data in charts are depicted as mean ± standard deviation of ≥3 independent experiments.

**Supplementary Figure 9.** ***Dll1*-/*Mef2C*-based MAB priming results in reduced fibrosis and increased muscle performance.** **(a)** Analysis of fibrotic scars in *tibialis anterior* muscles at 4 weeks post-injection. Masson’s trichromic staining reveals reduction in size and number of the fibrotic scars in the dystrophic mice injected with Ad-mock-, Ad-*Dll1*- or Ad-*Mef2C*-primed MABs. Double priming results in further reduction of fibrosis, as compared to single priming. Scale bar indicates 100μm. **(b)** Functional outcome at 8 weeks post-injection. Similarly to 4 weeks post-injection, both gait and treadmill analyses show functional improvement in mice injected with murine MABs primed with Ad-mock, or Ad-*Dll1*, or Ad-*Mef2C*. Combined priming with Ad-*Dll1* and Ad-*Mef2C* results in increased functional outcome of injected dystrophic mice as compared to single priming. *, P<0.05 vs sham; §, P<0.05 vs Ad*-Dll1* and vs Ad-*Mef2C* (n=3 mice/group).

**Supplementary Figure 10.** **RT-PCR evaluation of off-target engraftment of double-primed GFP^+^ murine MABs at 8 weeks post-injection.** Filter organs show almost undetectable levels of GFP. +, 10^5^ GFP^+^ MABs; -, RT- control. Similar results have been obtained from all conditions of murine MAB injection (data not shown).

**Supplementary Figure 11.** **Beneficial effects of combined priming of human MABs with *DLL1* and *MEF2C* on *in vivo* engraftment and regeneration.** **(a)** Immunofluorescence staining for human-specific sarcomeric ACTININa on *gastrocnemius* muscle sections. Depicted panels refer to the panels shown in Figure 2f. Scale bar indicates 100μm. **(b-c)** At 4 weeks after intra-arterial injection, priming of GFP^+^ human MABs with either Ad-*DLL1* or Ad-*MEF2C* results in higher rates of GFP^+^/hDYS^+^ fibers in *gastrocnemius* muscles, as assayed by immunofluorescence staining. Moreover, combined priming with both vectors results in higher rates of engrafted and regenerated fibers, as compared to single priming. This is confirmed by qPCR **(c)** analysis on *tibialis anterior* muscles. Expression levels are reported in arbitrary units (AU) as fold change vs Ad-mock. *, P<0.05 vs sham; §, P<0.05 vs Ad*-Dll1* and vs Ad-*Mef2C* (n=5 mice/group) Data in charts are depicted as mean ± standard deviation of ≥3 independent experiments.

**Supplementary Figure 12.** **Tamoxifen-driven *Dll1* knockout in flx cells and muscles.** **(a-b)** PCR analysis shows efficient *Dll1^flx^* removal from genomic DNA of flx AP^+^ MABs isolated from the skeletal muscle **(a)**, as well as of *tibialis anterior* (TA) and *gastrocnemius* (GCN) muscles of flx mice **(b)**. **(c)** WB analysis reveals downregulation of Dll1 protein levels after tamoxifen addition to flx cells and muscles. **(d-e)** Characterization of flx AP^+^ MABs. **(d)** After sorting, flx MABs stain extensively for AP enzymatic assay. **(e)** Antigen profiling confirms the characteristics of AP^+^ murine MABs; light grey line, isotype; dark grey line, flx AP^+^ MABs (depicted are the results obtained from 3 clones, isolated from 3 transgenic mice).

**Supplementary Figure 13. *Dll1* knockout in homing MABs results in impairment of engraftment and regeneration of dystrophic muscles.** **(a)** At 8 weeks after bilateral intra-arterial injection, tamoxifen-treated, hence *Dll1*-knockout, GFP^+^ MABs display reduced engraftment and regeneration levels in *tibialis anterior* muscles of injected dystrophic muscles, as revealed by WB analysis. **(b)** Analysis of fibrotic scars in *tibialis anterior* muscles at 8 weeks post-injection. Masson’s trichromic staining reveals increase in size and number of the fibrotic scars in the dystrophic mice injected with tamoxifen-treated flx MABs, as compared to vehicle control. Scale bar indicates 100μm. **(c)** Functional assessment of conditional *Dll1* knockout on MAB regeneration of dystrophic muscles at 4 weeks after bilateral injection, by means of gait analysis and treadmill assay. Data points depict the value of each assayed mouse, bars indicate the average values; *, P<0.05 vs vehicle-treated flx MABs (n=5 mice/group).

**Supplementary Figure 14. Effects of tamoxifen-driven *Dll1* knockout in flx satellite cells and uninjured skeletal muscles.** **(a)** Immunofluorescence staining shows reduction of *Pax7*-expressing cells and increase of cells differentiating into myocytes or myotubes after removal of Dll1 in flx satellite cells under proliferative conditions *in vitro*. Scale bar indicates 100μm. In **(b)** are reported the quantitative analyses relative to the staining; *, P<0.05 vs vehicle control (n=3 clones from 3 transgenic mice); fusion index, % nuclei incorporated into MyHC^+^ structures. **(c)** Tamoxifen-treated *Dll1*-knockout muscles do not show significant apparent changes in morphology or in fibrosis in the absence of induced damage as compared to vehicle control, as assayed by Masson’s trichromic staining on *gastrocnemius* muscle sections (here reported) or by H&E staining (data not shown). Scale bar indicates 100μm.
